# Dietary Fatty-Acid Quality, Omega-3 and Omega-6 Intake, and Food Sources Among 18–22-Year-Old Students at a UK University (2016)

**DOI:** 10.3390/foods15213970

**Published:** 2026-11-05

**Authors:** Antonio Peña-Fernández, Guillermo Torrado Durán, M. Ángeles Peña Fernández

**Affiliations:** 1 Area of Legal and Forensic Medicine, Department of Surgery, Medical and Social Sciences, Faculty of Medicine and Health Sciences, University of Alcalá, Ctra. Madrid-Barcelona, Km. 33.6, 28871 Alcalá de Henares, Spain; 2 Leicester School of Allied Health Sciences, De Montfort University, Leicester LE1 9BH, UK; 3 Department of Biomedical Sciences, Faculty of Pharmacy, University of Alcalá, Crta. Madrid-Barcelona Km. 33.6, 28871 Alcalá de Henares, Spain

**Keywords:** dietary fatty acids, emerging adulthood, university students, saturated fatty acids, polyunsaturated fatty acids, omega-3, omega-6, EPA, DHA, food sources

## Abstract

Emerging adulthood is a critical period for establishing independent dietary behaviours, yet detailed information on fatty-acid intake and food sources in university populations remains limited. This historical cross-sectional study characterised dietary fatty-acid quality in 111 students aged 18–22 years attending De Montfort University, Leicester, UK, using dietary data collected in early 2016. Habitual dietary intake was assessed using an expanded semi-quantitative food-frequency questionnaire processed with Nutritics. Total fat, major fatty-acid classes and selected individual fatty acids were examined, while principal food sources were reconstructed using food-composition data. The median total-fat, SFA, MUFA and PUFA intakes were 77.67, 26.87, 28.19 and 11.18 g/day, respectively. Although 85.6% of participants were at or below the UK population benchmark for total fat, 69.4% were above the SFA benchmark. The median EPA+DPA+DHA intake was 0.126 g/day, with 82.9% below the UK long-chain n-3 population benchmark. Major SFA sources included dairy foods, red and processed meat, and discretionary bakery/confectionery products, whereas the largest PUFA contributions arose from discretionary bakery/confectionery products and fried potato/vegetable preparations. These historical findings highlight the value of assessing fatty-acid composition and food sources alongside total-fat intake; however, confirmation in larger, contemporary university cohorts is warranted.

## 1. Introduction

Emerging adulthood represents an important nutritional transition between adolescence and established adult life. For many young adults, entry into university coincides with leaving the family home, assuming greater responsibility for food purchasing and preparation, managing financial constraints, and experiencing substantial changes in meal timing, social eating, alcohol consumption and food availability. These changes can disrupt previously established dietary routines and contribute to increasingly heterogeneous patterns of food consumption. Studies conducted among university populations, including UK students, have identified the coexistence of comparatively health-conscious dietary behaviours with patterns characterised by convenience foods, snacks, discretionary products and other energy-dense foods [1,2,3,4].

Dietary fat is particularly relevant within this transition because its nutritional significance cannot be adequately represented by total-fat intake alone. Contemporary dietary guidance increasingly emphasises fatty-acid composition and food source rather than considering all dietary fats as nutritionally equivalent [5,6,7,8]. Saturated fatty acids (SFAs), monounsaturated fatty acids (MUFAs), polyunsaturated fatty acids (PUFAs) and trans fatty acids have different metabolic and physiological properties, while the PUFA fraction encompasses nutritionally distinct n-6 and n-3 families.

Linoleic acid (LA; 18:2n-6) and α-linolenic acid (ALA; 18:3n-3) are essential fatty acids because endogenous synthesis is insufficient to meet physiological requirements [9,10]. LA is the principal dietary precursor within the n-6 family, whereas ALA represents the essential parent fatty acid of the n-3 pathway. Longer-chain derivatives include arachidonic acid (AA; 20:4n-6) and the long-chain n-3 fatty acids eicosapentaenoic acid (EPA; 20:5n-3), docosapentaenoic acid (DPA; 22:5n-3) and docosahexaenoic acid (DHA; 22:6n-3). Although ALA can be converted metabolically to EPA and subsequently to DPA and DHA, conversion is limited in humans, particularly at the later stages of the pathway [11,12,13]. Direct dietary intake of preformed long-chain n-3 fatty acids therefore remains nutritionally important [9,13].

EPA and DHA are incorporated into membrane phospholipids and contribute to the generation of bioactive lipid mediators, regulation of lipid metabolism and physiological processes relevant to cardiovascular and neurological function [7,9]. Nevertheless, interpretation of PUFA intake requires nuance. Earlier discussions frequently focused on the dietary n-6:n-3 ratio and sometimes characterised n-6 PUFAs as intrinsically undesirable. More recent evidence does not support such a simple interpretation. LA and other n-6 fatty acids should instead be considered in terms of absolute intake, overall dietary composition and the nutrients or foods that they replace [14,15].

A similarly food- and substitution-sensitive interpretation applies to SFAs. Reducing SFA intake does not produce an identical nutritional effect regardless of the replacement nutrient. The metabolic effects of replacing SFAs with unsaturated fatty acids, particularly PUFAs, differ from those of replacing SFAs with refined carbohydrates, and the food matrix through which SFAs is consumed may provide additional context [5,6,7,8]. Consequently, neither the total-fat intake nor any single fatty-acid ratio provides a complete assessment of dietary-fat quality.

UK dietary reference values reflect these distinctions. Current UK guidance retains population-average recommendations of approximately 35% of food energy for total fat, no more than 10% for SFAs, approximately 12% for cis-MUFAs, 6% for cis n-6 PUFAs and no more than 2% for trans fatty acids, alongside specific reference criteria for essential and long-chain n-3 fatty acids [16,17]. Importantly, these values do not all represent the same type of dietary reference. Population-average recommendations should not be interpreted as diagnostic thresholds for individual nutritional adequacy or deficiency.

The contemporary UK food environment also illustrates why individual fatty-acid classes require separate consideration. Industrial trans-fat exposure has fallen markedly following changes in food formulation, whereas SFA intake remains a public health concern, and oily fish consumption remains limited in substantial sections of the population [18,19]. National dietary surveillance provides essential population context, but broad national age categories may not fully represent the distinctive nutritional circumstances of university students undergoing the transition towards independent living.

Research specifically involving university students supports this distinction. Sprake et al., in a study of 1448 students attending five UK universities, identified substantial heterogeneity in dietary patterns, demonstrating that the university population cannot be regarded as nutritionally homogeneous [3]. More recent comparisons of UK and international university populations have similarly shown substantial variation in food and nutrient intake, including differences in SFA consumption [20]. Such observations are particularly relevant in culturally diverse university settings, where previous dietary practices interact with a shared local food environment.

Historical European studies have demonstrated substantial variation in SFA and trans-fat intake among young adults [21], while systematic evidence indicates considerable geographical and age-related variation in n-3 and n-6 PUFA intake across Europe [22,23]. In Dutch young adults, for example, the median SFA intake was approximately 12.9% of energy in analyses based on the 2003 food supply, while trans-fat intake was approximately 1.0% of energy before modelling the effects of food reformulation [21].

Recent evidence suggests that considerable heterogeneity persists even within university populations. A contemporary UK study involving university students reported a mean estimated EPA+DHA intake of approximately 473 mg/day, although the analytical sample was small and differed from the present cohort in age, participant selection and dietary methodology [24]. Such differences reinforce the importance of considering distributions, study design and population characteristics rather than comparing mean intakes in isolation.

The foods supplying individual fatty acids provide an additional dimension of dietary quality. Similar PUFA intakes, for example, may arise from diets rich in fish, nuts, seeds and vegetable oils or from diets in which a substantial proportion is supplied by fried foods and energy-dense discretionary products. This distinction may be particularly relevant during emerging adulthood, when convenience, cost, availability and increasing dietary independence can strongly influence food choice. Nutrient-level assessment without consideration of food sources may therefore obscure important differences in the overall nutritional quality of the diet.

The present investigation uses dietary data collected in early 2016 and therefore provides a historical characterisation of fatty-acid intake within a defined university population. Although these observations cannot be assumed to represent contemporary student diets, they provide a reference point for contextual comparisons with subsequent dietary surveys and developments in the UK food environment. Changes in food formulation, the availability of alternative dietary-fat sources and patterns of food consumption may have influenced fatty-acid exposure since the original assessments. Nevertheless, differences in sampling, dietary-assessment methods and food-composition databases must be considered when comparing studies, and the present cross-sectional data cannot establish temporal trends.

Thus, the present cohort offered an opportunity to investigate these questions in detail. Preliminary observations from this population were previously reported in conference proceedings format [25]; however, the present study provides a substantially expanded analysis incorporating a comprehensive fatty-acid profile, interpretation within an appropriate dietary-reference framework, adjusted demographic analyses with correction for multiple testing, and systematic food-source attribution.

Accordingly, the objectives of this study were to (i) characterise total and individual dietary fatty-acid intake among UK university students aged 18–22 years; (ii) examine the distribution of fatty-acid intakes in relation to relevant UK dietary reference criteria; (iii) investigate sex- and geographical-background-related variation after adjustment for relevant covariates; and (iv) identify the principal food-group contributors to SFA, MUFA, PUFA and trans-fat intake.

We hypothesised that examining fatty-acid composition and food sources would reveal aspects of dietary quality that were not apparent from total-fat intake alone.

## 2. Materials and Methods

### 2.1. Study Design and Participants

A historical cross-sectional analysis was undertaken using dietary data collected as part of a wider nutritional and biomonitoring study conducted at De Montfort University (DMU), Leicester, UK, during the 2015/2016 academic year.

Students aged 18–22 years were recruited using complementary campus-based approaches. These included brief presentations during scheduled teaching activities, announcements through the University’s virtual-learning environment, posters and study information materials, and communication through student and course representatives. Potential participants received written information explaining the study and were subsequently invited to attend face-to-face appointments. Recruitment procedures for the wider nutritional and biomonitoring study have been described previously [26].

Participation was voluntary, and written informed consent was obtained before any study procedure was undertaken. Eligibility for the wider study required participants to be DMU students aged 18–22 years and able to provide informed consent. For the present nutritional analysis, sufficiently complete and interpretable dietary information was additionally required to permit dietary processing and analysis.

The final analytical cohort comprised 111 participants.

This study was initiated during the 2015/2016 academic year, with planning and preparation of study materials beginning in late 2015. Formal ethical approval was granted by Chair’s Action of the Faculty Research Ethics Committee, Faculty of Health and Life Sciences, De Montfort University, on 11 January 2016 (Ref. 1674). Participant recruitment, written informed consent and all study procedures contributing data to the present analysis were undertaken after ethical approval. Data collection was concentrated between January and mid-March 2016 and was completed before the Easter vacation. Subsequent protocol amendments were approved through the institutional ethics process. This study was conducted in accordance with the Declaration of Helsinki.

### 2.2. Demographic Variables

Age and sex were recorded during the study assessment. Geographical background was derived from recorded continent of birth and classified as Africa, Asia, Europe or Other.

Geographical background was used exclusively as a broad descriptive variable and analytical covariate. It was not interpreted as a proxy for ethnicity, cultural identity, religion, socioeconomic circumstances or dietary acculturation.

Only two participants were classified in the Other category. Consequently, inferential analyses of geographical background were restricted to participants from Africa, Asia and Europe (*N* = 109), whereas the complete cohort (*N* = 111) was retained for the primary descriptive and sex analyses.

### 2.3. Dietary Assessment

Habitual dietary intake was assessed using an expanded semi-quantitative food-frequency questionnaire (FFQ). The core questionnaire structure was adapted from the EPIC-Norfolk FFQ, version 6 (CAMB/PQ/6/1205), whose underlying dietary-assessment methodology has been described previously [27]. Questionnaire development was also informed by the research group’s earlier published use of a semi-quantitative FFQ to estimate dietary intakes in young Spanish adults [28]. For the present cohort, the instrument was expanded to improve coverage of foods likely to be consumed within the geographically diverse university population in Leicester. The administered questionnaire contained more than 130 food and beverage entries and assessed usual consumption during approximately the four weeks preceding the assessment. The expanded instrument was not independently validated against a reference dietary-assessment method in this cohort.

Participants reported consumption using nine predefined frequency categories ranging from no or infrequent monthly consumption to multiple consumption occasions per day. Portion sizes were reported as small, medium or large relative to food-specific reference portions.

The questionnaire covered breads and cereals, vegetables, pulses and grains, fruit, nuts and seeds, meat and processed meat products, poultry, eggs, fish and seafood, dairy foods and alternatives, fats and oils, mixed dishes, discretionary foods, beverages and other commonly consumed foods. Additional or modified entries were incorporated to improve representation of foods likely to be consumed within the study population. These adaptations were intended to improve dietary capture rather than to assign particular foods to participants according to geographical background or infer culturally specific dietary behaviour.

In addition to the food-frequency tables, the questionnaire included supplementary questions addressing usual milk consumption, cooking fats and oils, selected dietary restrictions, fortified beverages and nutritional supplement use. The supplement section recorded frequency and dose information for broad supplement categories, including protein supplements, amino acids, vitamins, minerals and multivitamin/multimineral preparations. However, the questionnaire did not include a dedicated item specifically identifying fish oil, algal oil or other EPA/DHA-containing supplements. Consequently, the available questionnaire information did not permit a reliable, separate quantification of supplemental long-chain n-3 fatty-acid intake.

Completed FFQs were processed using Nutritics Research Edition version 5.7 (Nutritics Ltd., Dublin, Ireland). The analytical source variable represented weekly food consumption. Weekly food intake was converted to estimated daily intake by division by seven, with the original weekly variables retained separately during data processing.

### 2.4. Fatty-Acid Variables and Derived Indicators

The principal dietary variables were total fat, SFAs, MUFAs, PUFAs, total n-3 PUFAs, total n-6 PUFAs and trans fatty acids. Individual fatty acids comprised LA, ALA, AA, EPA, DPA and DHA.

Four additional compositional indicators were calculated:

PUFA:SFA ratio = total PUFA/total SFA;

n-6:n-3 ratio = total n-6 PUFA/total n-3 PUFA;

EPA+DHA = EPA + DHA;

EPA+DPA+DHA = EPA + DPA + DHA.

The PUFA:SFA and n-6:n-3 ratios were treated as descriptive indicators of fatty-acid composition and were not interpreted as clinical targets.

Aggregate PUFAs, total n-3 PUFAs and total n-6 PUFAs were retained from the corresponding native Nutritics nutrient variables, whereas LA, ALA, AA, EPA, DPA and DHA were extracted as separately reported individual fatty-acid estimates. The selected individual fatty acids were not intended to represent an exhaustive breakdown of the corresponding aggregate classes.

An additional participant-level reconciliation audit was undertaken to examine the numerical differences between aggregate fatty-acid measures and the sums of the reported individual components. The original aggregate values and individual fatty-acid estimates were reproduced from the source dataset. However, the available software documentation did not establish that the aggregate variables were calculated exclusively as the sum of the individual species reported in the export. Accordingly, these discrepancies were not treated as evidence of a specific computational error, and the original nutrient estimates were retained without retrospective adjustment.

### 2.5. Food Energy Excluding Ethanol

Percentage-energy variables were calculated relative to food energy excluding ethanol (EEE), consistent with the denominator convention underlying the relevant UK dietary reference values [16]. Alcohol-derived energy was excluded when calculating percentage-energy variables. For red wine, the ethanol content was assigned from the UK Composition of Foods Integrated Dataset (CoFID) [29] using food code 17-752 (“Wine, red”; 10.7 g alcohol/100 mL), assuming a specific gravity of 1.00. Weekly wine intake was converted to daily consumption once, ethanol intake was calculated from the corresponding CoFID alcohol concentration, and ethanol-derived energy was excluded from the denominator used to calculate the percentage-energy fatty-acid variables.

Two alternative ethanol concentration scenarios were retained as sensitivity analyses. Neither changed participant classification against the examined dietary reference criteria (Table S1). The CoFID value of 10.7 g alcohol/100 mL was therefore retained for the primary analysis.

### 2.6. Dietary Reference Framework

Dietary reference values (DRVs) were interpreted according to their intended population- or individual-level application rather than being treated as interchangeable thresholds of nutritional adequacy. For binary classifications relative to the prespecified dietary reference criteria, 95% confidence intervals for cohort proportions were calculated using the Wilson score method [30].

The UK population-average framework included approximately 35% of food energy for total fat, 10% for SFAs, 12% for *cis*-MUFAs, 6% for *cis* n-6 PUFAs and 2% for trans fatty acids [16]. Individual minimum intakes of 1% and 0.2% of food energy were applied to LA and ALA, respectively, in accordance with the UK dietary-reference framework [16].

Long-chain n-3 intake, expressed as EPA+DPA+DHA, was contextualised against the UK population benchmark of 0.45 g/day [16,17].

Population-average recommendations were not interpreted as individual diagnostic thresholds. The proportion of participants above or below these values was therefore used to describe the cohort distribution rather than to estimate individual clinical deficiency or inadequacy.

Particular caution was applied to MUFAs. The dietary variable available from the analytical dataset represented total MUFAs, whereas the UK population reference applies specifically to *cis*-MUFAs. The 12% energy value was consequently retained only as descriptive population context and was not used as an individual classification threshold. The 6% energy n-6 population reference was interpreted similarly as contextual rather than as a criterion of individual adequacy.

### 2.7. Food-Source Reconstruction

A separate food-source reconstruction was undertaken to identify the foods contributing to the observed fatty-acid profile. This reconstruction did not replace the original Nutritics-derived participant nutrient estimates, which remained the primary exposure variables. Instead, food-composition data from the UK CoFID [29] were used as a separate food-source attribution layer to characterise the food sources underlying those exposures.

The dietary database contained 441 distinct FoodName entries. Each entry was assessed for an appropriate food-composition match and assigned to one of 15 mutually exclusive food-source groups. The source taxonomy was developed specifically for fatty-acid attribution and therefore distinguished nutritionally relevant categories including dairy foods and alternatives; red and processed meat; poultry; fish and seafood; eggs; nuts and seeds; added fats and dressings; fried potato and vegetable preparations; sweet discretionary, bakery and confectionery products; mixed/composite dishes and sauces; and other food sources.

The food-source mapping comprised two distinct steps: assignment of individual FoodName entries to documented food-composition records, where available, and classification into 15 mutually exclusive food-source groups. A version-controlled crosswalk was used to retain information on the matching procedure and to identify uncertain correspondences. All 441 FoodName entries were assigned a source-group label, and all 9935 positive-consumption records were successfully linked to the classification system. Successful group assignment did not necessarily imply the availability of quantifiable fatty-acid composition data for every record. An additional targeted audit examined 20 food entries prioritised for potential mapping uncertainty, identifying three nominal matches, six conditional matches and 11 non-direct matches. The original food-composition assignments were retained without introducing unverified substitutions. Exclusion-based sensitivity analyses were performed to assess the influence of these uncertain matches on the principal reconstructed food-source contributions. These analyses were treated as influence diagnostics rather than corrected estimates of dietary intake.

The initial food-composition matching and food-source group assignments were performed by the corresponding author (A.P.-F.). The completed mapping was subsequently reviewed by a clinical research dietitian who had not participated in the initial assignments. This constituted a secondary quality-control review rather than two independently conducted parallel mappings. Food entries for which a direct nutritional equivalence could not be established were identified as uncertain. These entries were retained in the primary reconstruction, and their influence was assessed through exclusion-based sensitivity analyses without introducing unverified replacement food-composition values.

Reconstructed nutrient totals were compared with the participant-level nutrient estimates to determine whether quantitative food-source attribution was sufficiently reliable for each fatty-acid measure. Agreement was considered sufficient for quantitative attribution of SFAs, MUFAs, PUFAs and trans fatty acids. For total n-3 and n-6 PUFAs and individual fatty acids, the reconstruction was considered suitable only for examining broad source patterns; consequently, exact quantitative food-source contributions were not reported for these measures.

Quantitative agreement between reconstructed and Nutritics-derived nutrient estimates was evaluated at the participant level (*N* = 111). Lin’s concordance correlation coefficient (CCC) [31] and Bland–Altman 95% limits of agreement [32] were used to assess agreement; 95% confidence intervals for the CCC were estimated from 2000 participant-level bootstrap resamples [33]. Mean bias (reconstructed minus Nutritics), mean absolute error (MAE), root mean square error (RMSE), and the ratio of reconstructed to Nutritics-derived cohort-level nutrient totals were also calculated. The agreement results are presented in Supplementary Table S2. The bootstrap agreement analysis used a fixed seed (20261021), while the food-group contribution analysis used a separate fixed seed (20260928).

For each fatty-acid class, the percentage contribution of food group *g* was calculated using the ratio-of-sums approach:

Contribution g (%) = 100 × [Σ intake from food group g/Σ intake from all food groups].

Uncertainty in these contribution estimates was quantified using 2000 participant-level bootstrap resamples; percentile-based 95% confidence intervals were obtained from the bootstrap distribution [33].

### 2.8. Statistical Analysis

The original statistical analyses were conducted in R version 4.4.2, whereas the additional concordance and food-source influence analyses were conducted in R version 4.3.1 (R Foundation for Statistical Computing, Vienna, Austria) [34]. Continuous variables were summarised using the mean ± standard deviation (SD) and median [interquartile range (IQR)] as appropriate, while categorical variables were reported as *n* (%). Because several individual fatty-acid distributions were strongly right-skewed, both the mean and median values were retained where their joint interpretation was informative.

Sex-related differences were examined using multivariable regression models. Fatty-acid outcomes were transformed using the natural logarithm, and models included sex, age, geographical background and log-transformed total energy intake, consistent with established approaches to energy adjustment in nutritional epidemiology [35]. HC3 heteroscedasticity-consistent standard errors were used for inference [36]. Regression coefficients were exponentiated and expressed as adjusted percentage differences between males and females with 95% confidence intervals.

The primary sex analysis used the complete cohort (*N* = 111). A prespecified sensitivity analysis excluded the two participants in the Other geographical background category, producing an analytical sample of *N* = 109.

Multiplicity was addressed using the Benjamini–Hochberg false-discovery-rate (BH-FDR) procedure across the prespecified family of fatty-acid outcomes [37]. Interpretation emphasised adjusted effect estimates, 95% confidence intervals and FDR-adjusted *q*-values rather than isolated nominal *p*-values.

Geographical background differences were examined among participants classified as African, Asian or European (*N* = 109). Global robust tests were performed first, followed by pairwise comparisons where appropriate. BH-FDR correction was applied to the corresponding inferential families.

This study is reported in accordance with the principles of the STROBE statement for observational cross-sectional studies [38].

## 3. Results

### 3.1. Dietary Fat and Fatty-Acid Profile

**Figure 1:**
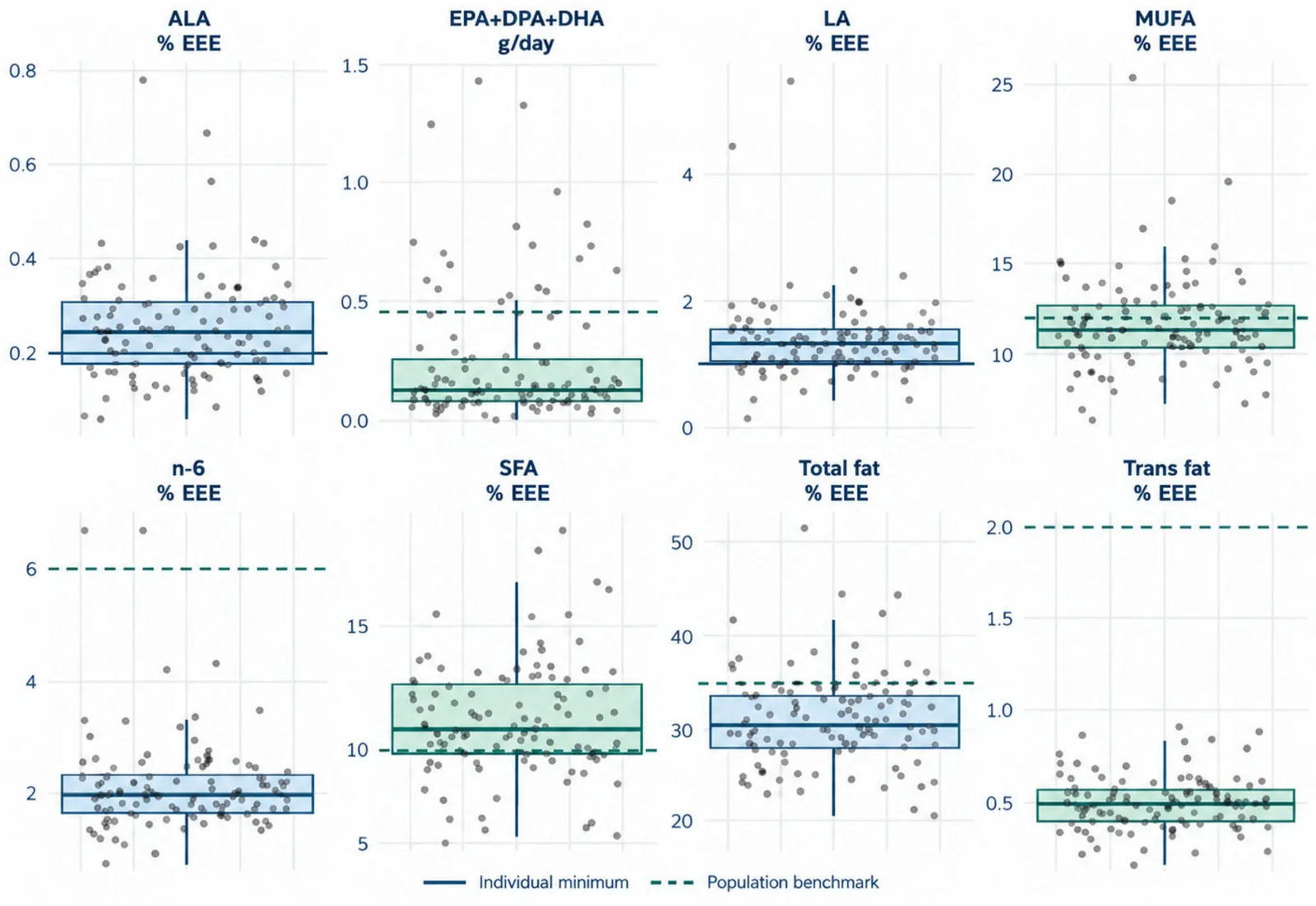
Distribution of selected dietary fat and fatty-acid measures among university students aged 18–22 years (*N* = 111). Individual observations are shown together with boxplots representing the median and interquartile range. Horizontal reference lines indicate the applicable UK individual minimum or population-average dietary reference value [16,17]. Population-average values are shown for contextual comparison and should not be interpreted as individual requirements. The MUFA panel represents total MUFAs, whereas the corresponding UK reference applies specifically to cis-MUFAs and is therefore shown for descriptive context only. EEE, energy excluding ethanol; SFAs, saturated fatty acids; MUFAs, monounsaturated fatty acids; LA, linoleic acid; ALA, alpha-linolenic acid; EPA, eicosapentaenoic acid; DPA, docosapentaenoic acid; DHA, docosahexaenoic acid.

The final analytical cohort comprised 111 participants, including 78 females (70.3%) and 33 males (29.7%). The mean age was 20.4 ± 1.2 years. Geographical background was classified as Africa in 41 participants (36.9%), Asia in 41 (36.9%), Europe in 27 (24.3%) and Other in 2 (1.8%). All 111 participants were retained in the descriptive and primary sex analyses, whereas geographical background comparisons included 109 participants after exclusion of the two individuals in the sparsely represented Other category. The complete dietary fatty-acid profile of the 111 participants is presented in Table 1, while distributions of selected nutritionally relevant measures are shown in Figure 1. Additional descriptive statistics are provided in Supplementary Table S3.

The median total-fat intake was 77.67 g/day [IQR 54.72–106.82], compared with a mean of 86.91 ± 45.35 g/day. The median SFA intake was 26.87 g/day [19.80–36.07], with a mean of 31.10 ± 16.93 g/day. The corresponding median intakes were 28.19 g/day [21.60–38.64] for MUFAs and 11.18 g/day [8.34–16.65] for PUFAs.

The median total n-3 PUFA intake was 1.336 g/day [0.807–2.016], whereas the median total n-6 PUFA intake was 5.137 g/day [3.469–7.060]. The median trans-fat intake was 1.178 g/day [0.827–1.585].

For the individual essential fatty acids, the median LA intake was 3.067 g/day [2.250–4.682], while the median ALA intake was 0.577 g/day [0.401–0.866].

Intakes of the long-chain n-3 fatty acids EPA, DPA and DHA were substantially lower than those of LA and ALA. The median EPA, DPA and DHA intakes were 0.035, 0.026 and 0.062 g/day, respectively. Consequently, the median combined EPA+DHA intake was 0.098 g/day, and the median EPA+DPA+DHA intake was 0.126 g/day.

### 3.2. Fatty-Acid Indicators and Dietary Reference Context

Fatty-acid quality indicators and the cohort distribution in relation to the prespecified UK dietary reference framework are summarised in Table 2; further details are provided in Supplementary Table S4.

Total fat accounted for a mean of 30.99 ± 5.18% of food energy, with a median of 30.57% [28.24–33.69]. Overall, 95 of 111 participants (85.6%) were at or below the UK population-average benchmark of 35% of food energy.

The pattern differed substantially for SFAs. SFAs accounted for a mean of 11.13 ± 2.40% of food energy, and 77 participants (69.4%) had values above the 10% energy population benchmark.

The mean MUFA and n-6 PUFA intakes were 11.71 ± 2.58% and 2.12 ± 0.87% of food energy, respectively. As specified in the dietary-reference framework, the corresponding population values were used for descriptive context only and not for individual classification.

For the essential fatty acids, 89 participants (80.2%) met the individual LA minimum of 1% of food energy, whereas 22 (19.8%) were below this criterion. For ALA, 75 participants (67.6%) met the individual minimum of 0.2% of food energy, leaving 36 participants (32.4%) below the criterion.

The median PUFA:SFA ratio was 0.405 [0.334–0.506], while the median n-6:n-3 ratio was 4.030 [3.164–4.983].

Long-chain n-3 intake showed one of the clearest departures from the reference context. The median EPA+DPA+DHA intake was 0.126 g/day [0.081–0.254], whereas the mean was 0.241 ± 0.280 g/day, indicating substantial positive skew. Only 19 participants (17.1%) were at or above the 0.45 g/day UK population benchmark, while 92 (82.9%) were below it.

In contrast, the trans-fat intake was low. Trans fatty acids accounted for a mean of 0.486 ± 0.150% of food energy, and all participants were below the UK population benchmark of 2% of food energy.

### 3.3. Sex Differences in Fatty-Acid Intake

In the primary multivariable analysis (*N* = 111), sex was associated with several broad fatty-acid classes after adjustment for age, geographical background and total energy intake (Table S5).

Adjusted total-fat intake was 10.5% lower in males than in females (95% CI: −17.0 to −3.6%; *p* = 0.004; *q* = 0.045). SFA intake was 13.7% lower in males (95% CI: −22.1 to −4.3%; *p* = 0.005; *q* = 0.045), and PUFA intake was 16.3% lower (95% CI: −26.5 to −4.7%; *p* = 0.008; *q* = 0.045).

The adjusted difference for MUFAs was −9.0% in males, but this association did not remain statistically significant after FDR correction (*p* = 0.044; *q* = 0.148). ALA intake was estimated to be 22.2% higher in males (95% CI: 1.3–47.5%; *p* = 0.036), but this difference similarly did not survive multiplicity correction (*q* = 0.148).

No FDR-significant sex differences were identified for total n-3, total n-6, trans fatty acids, LA, AA, EPA, DPA or DHA.

In the sensitivity analysis excluding the two participants in the Other geographical background category (*N* = 109), effect estimates remained directionally consistent, although total fat, SFAs and PUFAs no longer met the FDR-adjusted significance threshold (Table S6). Concordance between the primary and sensitivity analyses is summarised in Table S7.

### 3.4. Geographical Background Comparisons

No global geographical background comparison remained statistically significant after BH-FDR correction (Table S8). Pairwise comparisons among participants classified as African, Asian and European similarly produced no FDR-significant differences (Table S9).

Accordingly, although nominal variation was present for some fatty-acid measures, the adjusted analyses provided no robust evidence that fatty-acid intake differed according to the broad geographical background categories examined.

### 3.5. Food Sources of SFAs, MUFAs, PUFAs and Trans Fatty Acids

Participant-level agreement between reconstructed and original Nutritics-derived intake estimates was high for the four fatty-acid classes used in quantitative food-source attribution (Supplementary Table S2). Lin’s concordance correlation coefficients were 0.994 for SFAs, 0.991 for MUFAs, 0.985 for PUFAs and 0.979 for trans fatty acids. The corresponding cohort-level recovery percentages were 100.0%, 102.3%, 94.3% and 106.0%, respectively. Complete agreement statistics, including bootstrap confidence intervals, bias and error measures, are reported in Supplementary Table S2. These results supported the use of the reconstruction for broad fatty-acid class attribution while not establishing the nutritional equivalence of every individual food-composition match.

**Figure 2:**
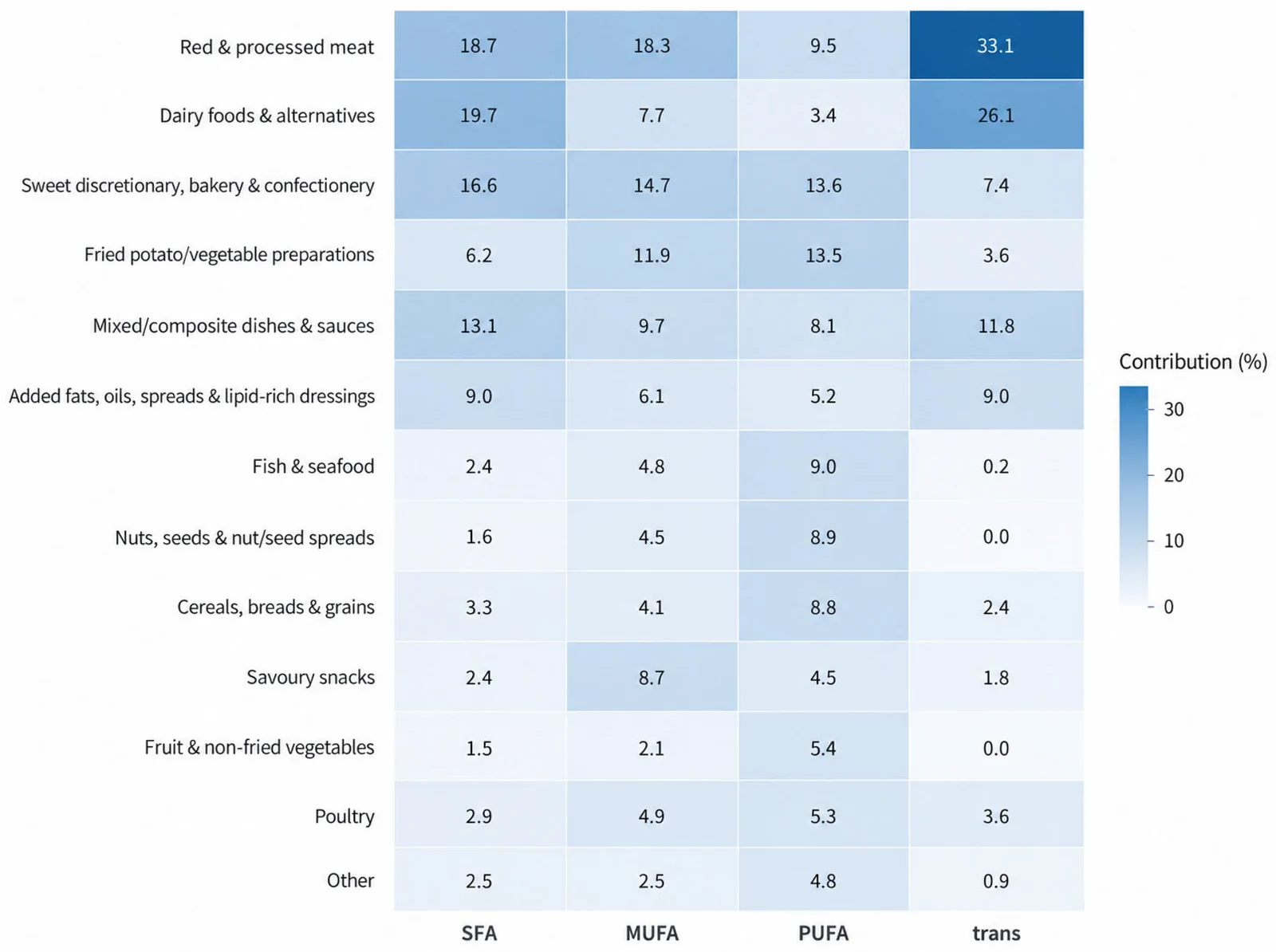
Relative contribution of food groups to dietary SFA, MUFA, PUFA and trans fatty-acid intake. Cell values represent the percentage contribution of each food group to reconstructed cohort-level intake of the corresponding fatty-acid class. Food groups contributing outside those displayed individually are combined as “Other”. SFAs, saturated fatty acids; MUFAs, monounsaturated fatty acids; PUFAs, polyunsaturated fatty acids.

The principal food-group contributions to SFA, MUFA, PUFA and trans-fat intake are presented in Table 3 and visualised in Figure 2. The complete food-group contribution profiles are provided in Table S10 and Figure S1.

SFA intake was distributed across several food categories. Dairy foods and alternatives contributed the largest share of reconstructed SFA intake (19.7%; 95% bootstrap CI: 17.2–22.0%). Red and processed meat contributed 18.7% (16.4–21.1%), while sweet discretionary, bakery and confectionery products accounted for a further 16.6% (13.9–19.7%).

Red and processed meat represented the largest reconstructed MUFA source, contributing 18.3% (16.1–20.5%). Sweet discretionary, bakery and confectionery products contributed 14.7% (12.3–17.3%), followed by fried potato and vegetable preparations at 11.9% (9.4–15.1%).

A different and potentially important pattern was observed for PUFAs. The largest contribution arose from sweet discretionary, bakery and confectionery products, which accounted for 13.6% (11.0–16.5%) of reconstructed PUFA intake. Fried potato and vegetable preparations contributed a similar 13.5% (10.0–17.6%), while red and processed meat accounted for 9.5% (8.1–11.1%).

Trans-fat sources differed markedly from those of PUFAs. Red and processed meat accounted for 33.1% (29.9–36.5%) of reconstructed trans-fat intake and dairy foods and alternatives for 26.1% (23.1–28.9%). Mixed/composite dishes and sauces contributed a further 11.8% (10.3–13.4%).

## 4. Discussion

### 4.1. Principal Findings

This study identified a marked contrast between total-fat quantity and fatty-acid quality in young university students. Although total-fat intake was generally consistent with the UK population benchmark, the SFA intake was comparatively high, the ALA intake was below the individual minimum in approximately one-third of participants, and the typical long-chain n-3 intake was low. Food-source reconstruction provided an additional dimension to this profile: substantial PUFA intake arose from sweet discretionary, bakery and confectionery products and fried potato and vegetable preparations rather than exclusively from foods conventionally promoted as sources of unsaturated fat. Together, these findings indicate that assessment of total-fat intake alone can conceal important differences in both fatty-acid composition and the foods supplying those fatty acids.

More than four-fifths of participants were at or below the UK population-average benchmark for total fat, yet approximately seven in ten had SFA intakes above the corresponding population benchmark. At the same time, almost one-third of participants were below the individual ALA minimum, and typical direct intake of long-chain n-3 fatty acids was low. The median EPA+DHA intake was only 98 mg/day, and the median EPA+DPA+DHA intake was 126 mg/day, with 82.9% of participants below the UK population benchmark used for long-chain n-3 fatty acids.

The food-source analysis adds an important dimension to these nutrient-level findings. Major SFA sources included dairy foods, red and processed meat and discretionary bakery and confectionery products, while the two largest reconstructed contributors to PUFA intake were sweet discretionary, bakery and confectionery products and fried potato and vegetable preparations. Thus, a nutritionally favourable classification at the level of fatty-acid unsaturation did not necessarily imply that the foods supplying those fatty acids represented an equally favourable overall dietary pattern.

Together, these findings illustrate why dietary-fat quality should be evaluated simultaneously at the level of fatty-acid composition and food source.

### 4.2. Total Fat and SFAs: Quantity and Quality Tell Different Stories

The contrast between total fat and SFAs was particularly striking. The mean total-fat intake was approximately 31% of food energy, and 85.6% of participants were at or below the UK population-average benchmark of 35%. A conventional assessment focused predominantly on total-fat quantity could therefore suggest relatively little cause for concern.

SFAs produced a different interpretation. The mean intake represented 11.13% of food energy, and 69.4% of participants had values above the UK population-average benchmark of 10%. The finding is consistent with the broader observation that SFAs remain an important component of dietary-fat quality even in populations whose total-fat intake is not particularly high.

Recent UK national dietary surveillance provides contemporary context, indicating that SFA intake remains above recommended population levels in adults and confirming the continuing relevance of SFAs as a public health dietary concern [19].

Historical data in European young adults provide useful context. Temme et al. reported a median SFA intake of approximately 12.9% of energy among Dutch young adults when using 2003 food-composition data [21]. Although direct comparison is limited by differences in dietary methodology, food supply and study period, both studies indicate that SFAs can remain comparatively high even when overall dietary-fat exposure is not extreme.

Contemporary university data also demonstrate substantial between-population variability. Recent cross-cultural work comparing university students in the UK and United Arab Emirates reported higher absolute SFA intake among UK participants, with a mean difference of approximately 4.2 g/day [20]. This reinforces the importance of considering the local food environment when interpreting dietary-fat profiles in university populations.

Taken together, these comparisons suggest that the principal concern in this cohort was not the overall quantity of dietary fat but its fatty-acid composition, particularly the balance between SFAs and unsaturated fatty acids.

### 4.3. Food Sources Help Explain the SFA Profile

The source reconstruction provides additional context for this SFA exposure. Dairy foods and alternatives contributed approximately 19.7% of reconstructed SFAs, red and processed meat 18.7%, and sweet discretionary, bakery and confectionery products 16.6%.

This distribution is important because SFAs are not consumed in isolation. Dairy foods, meat products and bakery/confectionery foods differ substantially in protein, micronutrient, sodium, sugar and overall energy-density profiles, and the health implications of an SFA-containing food cannot necessarily be inferred from its SFA content alone. Contemporary dietary-fat research increasingly recognises the importance of the food matrix and replacement nutrients when considering associations between SFAs and health outcomes [5,6,7,8].

For dietary intervention, this means that simply advising students to “reduce saturated fat” may be less informative than identifying the foods responsible for a substantial proportion of exposure. The sizeable contribution from discretionary bakery and confectionery products is particularly relevant because reductions in these foods could modify SFA exposure while simultaneously influencing free sugars, energy density and other dimensions of diet quality.

### 4.4. PUFA Intake Illustrates the Importance of Food Source

The PUFA findings provide a clear example of why nutrient quantity alone may provide an incomplete picture of dietary quality.

PUFAs are conventionally regarded as a favourable replacement for SFAs within dietary guidance, particularly when the substitution occurs within an otherwise nutritionally appropriate diet [5,6,7,8]. However, the present source reconstruction showed that the largest contributors to PUFA intake were not fish, nuts or seeds but sweet discretionary, bakery and confectionery products (13.6%) and fried potato and vegetable preparations (13.5%).

This does not imply that the PUFAs contained in these foods become physiologically undesirable. Rather, it demonstrates that a favourable fatty-acid class can be delivered within foods that have very different overall nutritional characteristics.

Vegetable oils used in frying, bakery production and manufactured foods can provide substantial LA and other PUFAs [9,10]. Consequently, a higher PUFA intake may coexist with high energy density, refined carbohydrates, added sugars, sodium or repeated consumption of fried foods. Conversely, similar amounts of PUFAs obtained through nuts, seeds, fish or minimally processed plant foods occur within a substantially different dietary matrix.

This distinction is highly relevant when nutrient targets are translated into food-based advice. An intervention designed merely to increase PUFA intake without considering its sources could theoretically improve a nutrient-level measure while doing little to improve other dimensions of dietary quality and could potentially worsen them.

The present results therefore support integrating nutrient-based and food-based assessments: fatty-acid composition remains nutritionally relevant, but the foods supplying those fatty acids provide essential context for interpreting overall dietary quality.

### 4.5. Essential Fatty Acids: LA and ALA Show Different Patterns

The essential fatty acids LA and ALA showed notably different distributions.

Approximately 80.2% of participants met the individual LA minimum of 1% of food energy, leaving approximately one-fifth below this criterion. By comparison, only 67.6% met the individual ALA minimum of 0.2% of food energy, with 32.4% below it.

This difference is plausible in the context of contemporary food supplies. LA is widely distributed in commonly used vegetable oils and foods manufactured or prepared with those oils, whereas concentrated ALA sources are more restricted. Important dietary sources of ALA include flaxseed/linseed, chia, walnuts and selected vegetable oils [9,10].

The distinction also has physiological relevance. ALA is an essential fatty acid in its own right and serves as the precursor of longer-chain n-3 fatty acids. However, the capacity of humans to convert ALA to EPA is limited, and conversion through to DHA is considerably more restricted [11,12,13]. The conversion efficiency can additionally vary according to sex, background diet and metabolic factors [13].

The present data illustrate why total n-3 intake should therefore not be regarded as interchangeable with long-chain n-3 exposure. The median total n-3 intake was 1.336 g/day, whereas the median EPA+DHA intake was only 0.098 g/day. An aggregate “omega-3” value would consequently conceal a substantial difference between the intake of the parent essential fatty acid and direct intake of the longer-chain compounds.

### 4.6. Low Long-Chain n-3 Intake Was the Most Pronounced Fatty-Acid Gap

The clearest departure from the dietary reference context involved EPA, DPA and DHA.

The median EPA and DHA intakes were 35 and 62 mg/day, respectively. The median combined EPA+DHA intake was 98 mg/day, while inclusion of DPA increased the median long-chain n-3 intake to 126 mg/day.

These values are low when considered alongside European and international recommendations for long-chain n-3 fatty acids, although the exact numerical recommendations and the fatty acids included differ between authorities [22,23,39]. The UK population benchmark prespecified for the present analysis was 0.45 g/day for EPA+DPA+DHA [16,17], against which only 17.1% of participants were at or above the benchmark.

An especially informative feature of the distribution was the difference between the mean and the median. The mean EPA+DPA+DHA intake was 241 mg/day, almost twice the median of 126 mg/day. This positive skew indicates that a relatively small subgroup of higher consumers substantially increased the cohort mean.

This is nutritionally important because the mean alone would have overstated the intake experienced by the typical participant. In populations in which oily fish or other concentrated sources are consumed regularly by only a minority, the median intake and the complete distribution can be substantially more informative than the arithmetic mean.

European evidence supports considerable heterogeneity in the long-chain n-3 intake. Sioen et al. identified substantial variation in n-3 exposure across European populations and age groups [22], while Givens and Gibbs highlighted comparatively low EPA and DHA intakes in younger adults and the challenge of achieving the recommended intake where oily fish consumption is limited [23].

A recent UK university study provides an informative contemporary comparison [24]. In its analytical sample of 50 students, the mean estimated EPA+DHA intake was approximately 473 mg/day. This is substantially higher than both the mean and, particularly, the median observed in the present cohort.

However, the difference should not be interpreted as evidence that long-chain n-3 intake has necessarily increased over time. The recent study involved an older student sample, with a mean age of approximately 25 years, and used different dietary methodology and participant-selection criteria. It also excluded omega-3 supplement users and several health conditions and contained substantial representation of postgraduate and nutrition students [24]. Each of these characteristics could influence estimated dietary intake.

Rather than supporting a simple temporal comparison, the two studies illustrate the considerable interindividual and inter-study heterogeneity in long-chain n-3 exposure among UK university populations. They also emphasise the importance of reporting distributional statistics and population characteristics when evaluating EPA and DHA intake.

### 4.7. Food-Based Interpretation of Long-Chain n-3 Intake

Fish and seafood are the principal conventional concentrated dietary sources of EPA and DHA, although meat, eggs and fortified foods can contribute smaller amounts. Marine foods and, increasingly, algal products provide direct dietary sources of EPA and/or DHA and may therefore be particularly relevant for individuals with low fish consumption [9,23,40].

The recent literature also highlights increasing interest in alternative and sustainable n-3 sources, including algal oils and other innovative food sources [40]. Such alternatives may become increasingly relevant for individuals who consume little or no fish and for university food environments seeking to accommodate vegetarian, vegan, cultural and sustainability-related dietary preferences.

Quantitative food-source attribution for individual EPA, DPA and DHA was not undertaken because agreement between reconstructed and participant-level estimates was insufficient to support precise source percentages for these individual fatty acids. Accordingly, the present data indicate low typical long-chain n-3 intake but do not support precise quantification of the individual food sources contributing to that intake.

### 4.8. n-6 Fatty Acids and the n-6:n-3 Ratio

The median n-6:n-3 ratio was 4.03, but this ratio was treated as a descriptive compositional measure rather than a therapeutic target. A ratio can change through variation in either n-6 or n-3 intake and therefore does not identify which component is responsible for the observed value. Moreover, current evidence does not support characterising LA or n-6 PUFAs as intrinsically pro-inflammatory at usual dietary intakes [14,15]. In this cohort, the more informative finding was the low absolute intake of EPA, DPA and DHA rather than the n-6:n-3 ratio itself.

### 4.9. Trans Fat Reflects a Different Nutritional Transition

Trans-fat intake presented a markedly different pattern from SFAs and long-chain n-3 fatty acids.

The median trans-fat intake was 1.18 g/day, corresponding to a mean of approximately 0.49% of food energy, and every participant was below the UK 2% energy population benchmark.

Historical comparison provides useful context. Temme et al. estimated trans-fat intake among Dutch young adults at approximately 1.0% of energy using food-composition information representing the 2003 food supply, with modelling suggesting a reduction to approximately 0.8% following food reformulation [21]. The substantially lower percentage observed in the present cohort is compatible with the wider reduction of industrially produced trans fatty acids from European and UK food supplies [18,21].

The reconstructed sources further support this interpretation. Red and processed meat accounted for 33.1% of the reconstructed trans-fat intake and dairy foods and alternatives for 26.1%, while mixed/composite dishes and sauces contributed 11.8%.

The predominance of red and processed meat and dairy foods among reconstructed sources is also consistent with the reduced contribution of partially hydrogenated oils to the contemporary UK food supply, although the present dietary data did not distinguish individual trans-fatty-acid isomers or whether they originated from industrial or ruminant sources [18].

Trans fat therefore did not emerge as a major quantitative concern in this cohort. Instead, the findings provide an example of how changes in food manufacturing and formulation can alter not only total nutrient exposure but also the relative importance of its dietary sources.

### 4.10. Sex Differences and the Importance of Energy Adjustment

Adjusted analyses identified sex differences in several broad fatty-acid classes. Males had approximately 10.5% lower total-fat, 13.7% lower SFA and 16.3% lower PUFA intake than females after adjustment for age, geographical background and total energy intake, and these associations survived BH-FDR correction in the primary analysis.

These results should not be interpreted as simple differences in the absolute amount of food consumed. Males can consume more total energy and consequently greater absolute quantities of individual nutrients while having a lower proportional or energy-adjusted exposure. Adjustment for total energy is therefore particularly important when examining dietary composition [35].

The sensitivity analysis indicates that the observed sex-related differences require cautious interpretation. Although the estimated differences for total fat, SFAs and PUFAs remained directionally consistent after excluding the two participants in the Other geographical background category, none retained statistical significance following BH-FDR correction in the reduced sample (*N* = 109). The corresponding adjusted estimates were −9.75%, −13.64% and −15.20%, respectively, compared with −10.53%, −13.67% and −16.29% in the complete cohort. Thus, the magnitude and direction of these associations were broadly stable, whereas their multiplicity-adjusted statistical classification was sensitive to the analytical sample. These findings should therefore be interpreted as exploratory evidence of possible sex-related differences rather than definitive associations.

Biological sex differences in fatty-acid metabolism, including ALA conversion, have been reported in the literature [13]. However, the present study measured dietary intake rather than fatty-acid metabolism or circulating fatty-acid status. The observed sex differences should therefore be interpreted primarily as dietary differences rather than being attributed to metabolic mechanisms.

### 4.11. Geographical Background

No geographical background comparison remained statistically significant after FDR correction; however, this absence of statistical significance should not be interpreted as evidence of equivalence between groups. The relatively modest cohort size, unequal group sizes and use of broad geographical categories may have limited the statistical power to detect smaller differences. Consequently, the findings indicate that no robust associations were identified within the present sample, rather than demonstrating that fatty-acid intakes were equivalent across geographical background groups.

This negative finding is important because nominal differences between participants classified as African, Asian and European could otherwise invite overinterpretation. Continent of birth is an extremely broad descriptor and cannot capture ethnicity, religion, cultural identity, socioeconomic circumstances, country-specific food traditions, duration of residence in the UK or dietary acculturation.

University attendance itself may also alter dietary behaviour. Migration, exposure to a shared food environment, financial constraints and greater dietary independence can modify previously established eating patterns [4,41].

The present data therefore provide no robust basis for attributing particular fatty-acid profiles to the broad geographical groups examined. More detailed investigation of migration history, cultural food practices and duration of residence would be required to address such questions appropriately.

### 4.12. Implications for University Food Environments

The combined findings have an important practical implication: a generic message to “reduce total fat” would be poorly targeted to this cohort.

Most participants were already at or below the UK total-fat population benchmark. The more relevant nutritional issues concerned fatty-acid composition, particularly SFA exposure and low long-chain n-3 intake, together with the foods supplying those fatty acids.

Food-based strategies in university settings could therefore focus on improving access to nutritionally appropriate sources of unsaturated fat, including nuts, seeds and suitable vegetable oils; facilitating regular oily fish consumption where acceptable; and providing appropriate fortified or algal alternatives for students who do not consume fish [40]. At the same time, reducing reliance on discretionary bakery and confectionery products and fried foods as major sources of dietary fat could improve fatty-acid quality alongside other aspects of the diet.

Dietary interventions should therefore aim to improve both the fatty-acid profile and the nutritional quality of the foods through which those fatty acids are consumed. University campuses provide a particularly relevant setting for such interventions because food purchasing and preparation habits are still being established, while institutional catering, pricing and availability can directly influence dietary choice [4].

### 4.13. Strengths and Limitations

This study has several strengths. The narrow age range of 18–22 years allowed focused examination of emerging adulthood without the substantial age heterogeneity inherent in many adult dietary surveys. This is particularly relevant because the transition into university life represents a distinctive period of increasing dietary independence.

The analysis also extended considerably beyond total fat and SFAs. Examination of MUFAs, PUFAs, total n-3 and n-6 fatty acids and individual LA, ALA, AA, EPA, DPA and DHA enabled important differences within the overall dietary-fat profile to be identified. In particular, the contrast between total n-3 and direct long-chain n-3 intake would have been obscured by a less detailed nutrient assessment.

A further strength was the explicit distinction between different types of dietary reference values. Population-average recommendations were not converted into individual deficiency thresholds, and the analytical mismatch between total MUFAs and the UK *cis*-MUFA population reference was explicitly acknowledged.

The separate food-source reconstruction represents another important strength. Participant-level Nutritics estimates remained the primary nutrient exposure measures, while the CoFID-based reconstruction was used specifically for food-source attribution. Complete mapping of the 441 FoodName entries and successful reconciliation of all 9935 positive-intake records provided a transparent and systematic framework for food-source attribution. Quantitative attribution was restricted to fatty-acid classes for which agreement between reconstructed and participant-level estimates was considered sufficient, rather than extending exact percentage claims to less well-reconciled individual fatty acids.

The mapping audit distinguished direct food-composition matches from approximate matches involving related foods, composite dishes or recipes. A targeted quality-control assessment was additionally undertaken for 20 food entries considered particularly relevant to mapping uncertainty. These entries were retained in the original reconstruction, and exclusion-based sensitivity analyses were conducted to quantify their influence on the principal food-source contributions. Such exclusions were treated as influence diagnostics rather than alternative estimates of actual dietary intake.

Participant-level bootstrap resampling additionally allowed uncertainty around food-source contributions to be quantified rather than presenting source percentages as fixed values.

Finally, inferential analyses incorporated multivariable adjustment, HC3 robust inference, BH-FDR multiplicity correction and sensitivity analysis. These procedures reduce the risk of overinterpreting isolated nominal *p*-values, particularly in a relatively modest cohort with multiple fatty-acid outcomes.

Several limitations should nevertheless be acknowledged. First, the cross-sectional design precludes causal inference and does not establish whether the observed dietary behaviours persisted beyond the period of data collection.

Second, dietary exposure was estimated using an FFQ. FFQs are appropriate for characterising habitual intake but remain susceptible to recall error, portion size misclassification and systematic under- or over-reporting [35]. Detailed fatty-acid estimates may additionally be affected by uncertainty in product formulation, recipes and cooking oils.

The adapted FFQ was not independently validated in the present cohort, and the interpretation of individual fatty-acid estimates may be affected by incomplete food-composition data and differences between aggregate and species-specific nutrient variables. In addition, discrepancies between aggregate fatty-acid classes and the sums of selected individual fatty acids were confirmed through participant-level auditing. Their precise origin could not be established from the available Nutritics documentation and may reflect differences in nutrient variable definitions or food-composition coverage. These discrepancies should not be interpreted as quantified amounts of missing fatty acids or attributed to a specific software calculation failure without further evidence. Furthermore, although the FFQ recorded general supplement use, it did not specifically identify fish oil, algal oil or other EPA/DHA-containing supplements, preventing reliable estimation of supplemental long-chain n-3 intake. Consequently, total EPA and DHA intake may have been underestimated among participants using such products. These limitations should be considered when interpreting the reported long-chain n-3 intake distributions and comparisons with dietary reference values.

Third, this study was conducted at a single UK university and was not designed to provide nationally representative prevalence estimates. The proportions of participants above or below particular reference values therefore describe this cohort and should not be extrapolated directly to all UK university students.

Fourth, the data were collected during the 2015/2016 academic year. The UK food supply and university food environment have subsequently evolved, including reformulation of manufactured foods and increased availability of plant-based products. Absolute food-source contributions in a contemporary cohort may therefore differ. Nevertheless, the historical dataset remains scientifically informative as a well-characterised snapshot of fatty-acid exposure during emerging adulthood and permits comparison with both earlier and more recent studies.

Furthermore, the modest sample size, volunteer-based recruitment and limited representation of a single university population constrain external validity. The observed intake distributions and food-source contributions cannot be assumed to represent other UK universities, international university populations or students in the current food environment. The geographical background analyses may also have been underpowered to identify modest associations, particularly after adjustment for multiple comparisons.

Fifth, reconstruction of food sources inevitably involves uncertainty, particularly for branded, composite or recipe-based foods for which exact historical composition may not be available. This was addressed through systematic mapping, participant-level agreement assessment and restriction of exact quantitative source attribution to fatty-acid classes for which agreement was considered sufficient.

Finally, this study assessed dietary intake rather than biological fatty-acid status. Circulating or tissue biomarkers would provide complementary information concerning absorption, endogenous metabolism and longer-term fatty-acid status, particularly for EPA and DHA. Future studies integrating dietary assessment with fatty-acid biomarkers would therefore provide a more complete characterisation of long-chain n-3 status during emerging adulthood.

## 5. Conclusions

Dietary-fat quality in this cohort of young university students could not be adequately characterised by total-fat intake alone. Although 85.6% of participants were at or below the UK population-average benchmark for total fat, 69.4% were above the corresponding SFA benchmark. Approximately one-third were below the individual ALA minimum, while 82.9% had long-chain n-3 intakes below the UK population benchmark applied in this study.

Long-chain n-3 exposure was particularly low in the typical participant, with median EPA+DHA and EPA+DPA+DHA intakes of 98 and 126 mg/day, respectively. The marked difference between the median and the mean long-chain n-3 intake also showed that cohort means can obscure low typical exposure when a minority of participants consume substantially higher amounts.

Food-source reconstruction provided information that nutrient quantities alone could not reveal. Dairy foods, red and processed meat, and sweet discretionary, bakery and confectionery products were major contributors to SFAs, whereas sweet discretionary, bakery and confectionery foods and fried potato and vegetable preparations were the two largest reconstructed contributors to PUFAs. These findings demonstrate that a nutritionally favourable fatty-acid class does not necessarily originate from an equally favourable overall food source.

Dietary strategies for emerging adults should therefore focus less on total-fat reduction alone and more on improving fatty-acid composition and the foods through which fatty acids are consumed. In this cohort, the most relevant priorities were addressing SFA exposure, increasing the intake of preformed long-chain n-3 fatty acids and promoting nutritionally appropriate sources of unsaturated fatty acids. University food environments may provide an important setting in which to support these dietary improvements while independent eating habits are being established.

## Figures and Tables

**Table 1:** Dietary fat and fatty-acid intakes among university students aged 18–22 years (*N* = 111).

Measure	Unit	Mean ± SD	Median [IQR]	Range
Total fat	g/day	86.910 ± 45.354	77.665 [54.720–106.823]	23.843–307.672
SFAs		31.098 ± 16.930	26.868 [19.802–36.073]	9.065–101.246
MUFAs		32.409 ± 16.227	28.192 [21.602–38.642]	8.638–106.113
PUFAs		13.377 ± 7.523	11.180 [8.343–16.649]	3.229–38.031
Total n-3		1.589 ± 1.022	1.336 [0.807–2.016]	0.386–5.283
Total n-6		5.832 ± 3.507	5.137 [3.469–7.060]	1.147–19.943
Trans fat		1.349 ± 0.789	1.178 [0.827–1.585]	0.290–5.047
LA		3.846 ± 2.443	3.067 [2.250–4.682]	0.132–16.266
ALA		0.699 ± 0.438	0.577 [0.401–0.866]	0.060–2.636
AA		0.089 ± 0.053	0.082 [0.058–0.102]	0.006–0.351
EPA		0.078 ± 0.102	0.035 [0.017–0.090]	0.002–0.512
DPA		0.035 ± 0.025	0.026 [0.017–0.045]	0.002–0.117
DHA		0.129 ± 0.160	0.062 [0.039–0.138]	0.002–0.818
Total fat	% energy excluding ethanol	30.990 ± 5.180	30.568 [28.238–33.694]	15.442–51.575
SFAs		11.129 ± 2.397	10.821 [9.830–12.640]	5.269–19.032
MUFAs		11.707 ± 2.581	11.309 [10.406–12.674]	6.358–25.227
PUFAs		4.775 ± 1.643	4.516 [3.808–5.181]	2.097–13.836
Total n-3		0.585 ± 0.332	0.511 [0.380–0.695]	0.120–2.647
Total n-6		2.119 ± 0.872	1.956 [1.652–2.326]	0.712–6.741
Trans fat		0.486 ± 0.150	0.483 [0.382–0.562]	0.143–0.910

Data are presented as mean ± standard deviation (SD), median [interquartile range (IQR)] and range. Percentage-energy values were calculated using food energy excluding ethanol. SFAs, saturated fatty acids; MUFAs, monounsaturated fatty acids; PUFAs, polyunsaturated fatty acids; LA, linoleic acid; ALA, alpha-linolenic acid; AA, arachidonic acid; EPA, eicosapentaenoic acid; DPA, docosapentaenoic acid; DHA, docosahexaenoic acid. Minimum and maximum values were calculated independently for each nutrient variable and do not necessarily correspond to the same participants. The individual fatty acids listed in this table represent selected species and should not be interpreted as an exhaustive decomposition of the aggregate fatty-acid classes.

**Table 2:** Dietary fatty-acid quality indicators and comparison with UK dietary reference values.

Indicator/Measure	Observed Cohort Statistic	UK Reference Value	Interpretation of Reference	Cohort Distribution Relative to Reference
PUFA:SFA	median 0.405 [0.334–0.506]		Descriptive indicator	
n-6:n-3	median 4.030 [3.164–4.983]		Descriptive indicator	
EPA+DHA	median 0.098 [0.061–0.209]		Descriptive indicator	
Total fat	mean 30.990 ± 5.180; median 30.568 [28.238–33.694]	35% energy excluding ethanol	Population-average recommendation	at/below population benchmark: 95/111 (85.6%; 95% CI 77.9–90.9)
Saturated fatty acids (SFAs)	mean 11.129 ± 2.397; median 10.821 [9.830–12.640]	10% energy excluding ethanol	Population-average recommendation	at/below population benchmark: 34/111 (30.6%; 95% CI 22.8–39.7)
MUFAs	mean 11.707 ± 2.581; median 11.309 [10.406–12.674]	12% energy excluding ethanol	Population-average recommendation	
n-6 PUFAs	mean 2.119 ± 0.872; median 1.956 [1.652–2.326]	6% energy excluding ethanol	Population-average recommendation	
Alpha-linolenic acid (ALA, n-3)	mean 0.254 ± 0.111; median 0.246 [0.178–0.307]	0.2% energy excluding ethanol	UK individual minimum	meets individual minimum: 75/111 (67.6%; 95% CI 58.4–75.6)
Linoleic acid (LA, n-6)	mean 1.392 ± 0.635; median 1.321 [1.063–1.544]	1% energy excluding ethanol	UK individual minimum	meets individual minimum: 89/111 (80.2%; 95% CI 71.8–86.5)
Long-chain n-3 PUFA (EPA+DPA+DHA)	mean 0.241 ± 0.280; median 0.126 [0.081–0.254]	0.45 g/day	Population-average recommendation	at/above population benchmark: 19/111 (17.1%; 95% CI 11.2–25.2)
Trans fatty acids	mean 0.486 ± 0.150; median 0.483 [0.382–0.562]	2% energy excluding ethanol	Population-average recommendation	at/below population benchmark: 111/111 (100.0%; 95% CI 96.7–100.0)

PUFA:SFA and n-6:n-3 ratios and EPA+DHA intake are presented as descriptive indicators and were not evaluated against a formal threshold. Population-average reference values are provided to contextualise the cohort distribution and should not be interpreted as individual requirements. The observed MUFA variable represents total MUFAs, whereas the UK population reference applies specifically to cis-MUFAs; this comparison is therefore descriptive only. SFAs, saturated fatty acids; MUFAs, monounsaturated fatty acids; PUFAs, polyunsaturated fatty acids; LA, linoleic acid; ALA, alpha-linolenic acid; EPA, eicosapentaenoic acid; DPA, docosapentaenoic acid; DHA, docosahexaenoic acid; CI, confidence interval. The observed n-6 variable represents total n-6 PUFAs, whereas the UK population reference applies specifically to cis n-6 PUFAs; this comparison is therefore descriptive only. UK dietary reference values were obtained from [16,17].

**Table 3:** Principal food-group contributors to dietary SFA, MUFA, PUFA and trans fatty-acid intakes.

Fatty Acid	Rank	Food Group	Contribution, %	Bootstrap 95% CI	Participants, *n* (%)
SFAs	1	Dairy foods and alternatives	19.7	17.2–22.0	109 (98.2)
SFAs	2	Red and processed meat	18.7	16.4–21.1	110 (99.1)
SFA	3	Sweet discretionary, bakery and confectionery	16.6	13.9–19.7	109 (98.2)
MUFAs	1	Red and processed meat	18.3	16.1–20.5	110 (99.1)
MUFAs	2	Sweet discretionary, bakery and confectionery	14.7	12.3–17.3	109 (98.2)
MUFAs	3	Fried potato/vegetable preparations	11.9	9.4–15.1	102 (91.9)
PUFAs	1	Sweet discretionary, bakery and confectionery	13.6	11.0–16.5	109 (98.2)
PUFAs	2	Fried potato/vegetable preparations	13.5	10.0–17.6	102 (91.9)
PUFAs	3	Red and processed meat	9.5	8.1–11.1	110 (99.1)
trans	1	Red and processed meat	33.1	29.9–36.5	110 (99.1)
trans	2	Dairy foods and alternatives	26.1	23.1–28.9	110 (99.1)
trans	3	Mixed/composite dishes and sauces	11.8	10.3–13.4	108 (97.3)

Food-group contributions were estimated using the cohort-level ratio-of-sums approach. Values represent the percentage contribution of each food group to reconstructed intake of the corresponding fatty-acid class. Confidence intervals were obtained by participant-level bootstrap resampling (2000 iterations). Participant *n* (%) indicates the number and proportion of participants with a positive reconstructed contribution to the corresponding fatty-acid class from foods assigned to that group. SFAs, saturated fatty acids; MUFAs, monounsaturated fatty acids; PUFAs, polyunsaturated fatty acids; CI, confidence interval.

## Data Availability

Aggregated data supporting the main tables and figures, supplementary analytical outputs, methodological inputs and R analysis code supporting this study are publicly available in Figshare at https://doi.org/10.6084/m9.figshare.34023501. Participant-level dietary records, raw FFQ/Nutritics/Access source files, identifiers, FoodName-level intake records and detailed food-composition crosswalks are not publicly available because they contain human-participant information and/or materials subject to ethical, institutional and data-protection requirements. Requests for additional de-identified information may be directed to the corresponding author and will be considered subject to applicable ethical and institutional requirements.

## Supplementary Materials

The following supporting information can be downloaded at https://www.mdpi.com/article/10.3390/foods15213970/s1, Table S1: Sensitivity of dietary-reference classifications to alternative assumptions for red wine ethanol content; Table S2: Participant-level agreement between reconstructed and Nutritics-derived dietary fatty-acid intake estimates (*N* = 111); Table S3: Detailed descriptive statistics for dietary fat and fatty-acid intakes in the study cohort (*N* = 111); Table S4: Detailed distribution of dietary fat and fatty-acid intakes relative to UK dietary reference values; Table S5: Adjusted differences in dietary fatty-acid intake between male and female participants (*N* = 111); Table S6: Sensitivity analysis of adjusted sex differences after exclusion of participants in the sparsely represented geographical background category (*N* = 109); Table S7: Comparison of effect estimates from the primary and sensitivity analyses of sex differences in dietary fatty-acid intake; Table S8: Global tests of differences in dietary fatty-acid intake according to geographical background (*N* = 109); Table S9: Pairwise comparisons of dietary fatty-acid intake according to geographical background (*N* = 109); Table S10: Food-group contributions to dietary SFA, MUFA, PUFA and trans fatty-acid intake; Figure S1: Complete food-group contribution profiles for dietary SFA, MUFA, PUFA and trans fatty acids. Contributions represent the percentage of reconstructed cohort-level intake attributable to each food group. SFA, saturated fatty acids; MUFA, monounsaturated fatty acids; PUFA, polyunsaturated fatty acids.
