# Supplementary material for: Dietary Fatty-Acid Quality, Omega-3 and Omega-6 Intake, and Food Sources Among 18–22-Year-Old Students at a UK University (2016)
Source: Foods. 2026 Nov 5;15(21):3970. doi: 10.3390/foods15213970 (PMC13762520; doi:10.3390/foods15213970)
Supplement: Supplementary file 1 [file foods-15-03970-s001.zip › foods-4634206-supplementary.pdf]

Table S1. Sensitivity of dietary-reference classifications to alternative assumptions for red-wine ethanol content.

Variable	Scenario	Alcohol g/100 mL	Median [IQR]	Reference	n classified	Classified, %	Changed vs primary
Total fat	low_8.7	8.7	30.549 [28.234–33.694]	35	95	85.6	0
SFA	low_8.7	8.7	10.821 [9.829–12.638]	10	34	30.6	0
MUFA	low_8.7	8.7	11.309 [10.405–12.674]	12			
n-6	low_8.7	8.7	1.956 [1.652–2.326]	6			
ALA	low_8.7	8.7	0.246 [0.178–0.307]	0.2	75	67.6	0
LA	low_8.7	8.7	1.321 [1.063–1.543]	1	89	80.2	0
trans	low_8.7	8.7	0.483 [0.382–0.562]	2	111	100	0
Total fat	legacy_9.6	9.6	30.558 [28.236–33.694]	35	95	85.6	0
SFA	legacy_9.6	9.6	10.821 [9.830–12.639]	10	34	30.6	0
MUFA	legacy_9.6	9.6	11.309 [10.405–12.674]	12			
n-6	legacy_9.6	9.6	1.956 [1.652–2.326]	6			
ALA	legacy_9.6	9.6	0.246 [0.178–0.307]	0.2	75	67.6	0
LA	legacy_9.6	9.6	1.321 [1.063–1.543]	1	89	80.2	0
trans	legacy_9.6	9.6	0.483 [0.382–0.562]	2	111	100	0
Total fat	primary_10.7	10.7	30.568 [28.238–33.694]	35	95	85.6	0
SFA	primary_10.7	10.7	10.821 [9.830–12.640]	10	34	30.6	0
MUFA	primary_10.7	10.7	11.309 [10.406–12.674]	12			
n-6	primary_10.7	10.7	1.956 [1.652–2.326]	6			

ALA	primary_10.7	10.7	0.246 [0.178–0.307]	0.2	75	67.6	0
LA	primary_10.7	10.7	1.321 [1.063–1.544]	1	89	80.2	0
trans	primary_10.7	10.7	0.483 [0.382–0.562]	2	111	100	0

Sensitivity analyses were performed using alternative assumed ethanol concentrations for red wine (8.7, 9.6 and 10.7 g/100 mL). The primary analysis used 10.7 g/100 mL. No participant classifications changed across the examined scenarios. MUFA and n-6 reference values were included for descriptive context only.

Table S2. Participant-level agreement between reconstructed and Nutritics-derived dietary fatty-acid intake estimates (*N* = 111).

Class	Lin CCC (95% CI)	Mean bias (g/day)	MAE (g/day)	RMSE (g/day)	Bland–Altman 95% LoA (g/day)	Recovery (%)
SFA	0.994 (0.990–0.996)	−0.007	1.235	1.841	-3.631 to 3.618	100.0
MUFA	0.991 (0.983–0.995)	+0.739	1.513	2.203	-3.347 to 4.824	102.3
PUFA	0.985 (0.978–0.990)	−0.759	0.886	1.275	-2.776 to 1.258	94.3
Trans	0.979 (0.961–0.986)	+0.080	0.128	0.165	-0.204 to 0.364	106.0

CCC, concordance correlation coefficient; CI, confidence interval; MAE, mean absolute error; RMSE, root mean square error; LoA, limits of agreement; SFA, saturated fatty acids; MUFA, monounsaturated fatty acids; PUFA, polyunsaturated fatty acids. Mean bias = reconstructed minus Nutritics-derived intake. Lin CCC 95% CIs were calculated using 2,000 participant-level bootstrap resamples (seed 20261021). Bland–Altman limits of agreement were calculated from the paired differences. Recovery (%) = 100 × (sum of reconstructed nutrient intakes / sum of Nutritics-derived nutrient intakes). Estimates are based on 111 participants. High agreement in cohort-level nutrient totals does not establish exact nutritional equivalence of individual food-composition matches or constitute an independent external validation of Nutritics.

Table S3. Detailed descriptive statistics for dietary fat and fatty-acid intakes in the study cohort (N = 111).

Variable	Metric	*N*	Missing	Mean	SD	Median	Q1	Q3	IQR	Min	Max	Skewness	IQR outliers
Total fat	g/day	111	0	86.910	45.354	77.665	54.720	106.823	52.103	23.843	307.672	1.847	6
	% total energy (provenance)	111	0	30.969	5.171	30.466	28.216	33.694	5.478	15.442	51.491	0.579	5
SFA	g/day	111	0	31.098	16.930	26.868	19.802	36.073	16.271	9.065	101.246	1.764	9
	% total energy (provenance)	111	0	11.122	2.392	10.821	9.815	12.634	2.819	5.269	18.999	0.408	4
MUFA	g/day	111	0	32.409	16.227	28.192	21.602	38.642	17.039	8.638	106.113	1.660	5
	% total energy (provenance)	111	0	11.699	2.578	11.309	10.397	12.674	2.277	6.358	25.186	1.622	6
PUFA	g/day	111	0	13.377	7.523	11.180	8.343	16.649	8.306	3.229	38.031	1.215	6
	% total energy (provenance)	111	0	4.772	1.642	4.516	3.806	5.181	1.375	2.097	13.814	2.202	6
Total n-3	g/day	111	0	1.589	1.022	1.336	0.807	2.016	1.209	0.386	5.283	1.377	6
	% total energy (provenance)	111	0	0.585	0.332	0.510	0.379	0.695	0.316	0.120	2.645	2.804	6
Total n-6	g/day	111	0	5.832	3.507	5.137	3.469	7.060	3.591	1.147	19.943	2.008	7
	% total energy (provenance)	111	0	2.117	0.872	1.956	1.652	2.326	0.675	0.712	6.740	2.993	6

Variable	Metric	*N*	Missing	Mean	SD	Median	Q1	Q3	IQR	Min	Max	Skewness	IQR outliers
Trans fat	g/day	111	0	1.349	0.789	1.178	0.827	1.585	0.758	0.290	5.047	1.932	8
	% total energy (provenance)	111	0	0.486	0.150	0.483	0.382	0.562	0.180	0.143	0.909	0.496	4
LA	g/day	111	0	3.846	2.443	3.067	2.250	4.682	2.431	0.132	16.266	2.212	6
	% total energy (provenance)	111	0	1.391	0.635	1.321	1.062	1.543	0.480	0.133	5.492	3.450	5
ALA	g/day	111	0	0.699	0.438	0.577	0.401	0.866	0.466	0.060	2.636	1.873	4
	% total energy (provenance)	111	0	0.254	0.111	0.246	0.178	0.307	0.129	0.061	0.780	1.611	3
AA	g/day	111	0	0.089	0.053	0.082	0.058	0.102	0.045	0.006	0.351	2.075	9
	% total energy (provenance)	111	0	0.034	0.017	0.031	0.021	0.040	0.020	0.003	0.083	0.941	6
EPA	g/day	111	0	0.078	0.102	0.035	0.017	0.090	0.073	0.002	0.512	2.321	13
	% total energy (provenance)	111	0	0.029	0.042	0.013	0.008	0.028	0.020	0.001	0.271	3.305	14
DPA	g/day	111	0	0.035	0.025	0.026	0.017	0.045	0.027	0.002	0.117	1.389	8
	% total energy (provenance)	111	0	0.013	0.008	0.011	0.008	0.015	0.007	0.001	0.061	2.669	7
DHA	g/day	111	0	0.129	0.160	0.062	0.039	0.138	0.099	0.002	0.818	2.259	18

Variable	Metric	*N*	Missing	Mean	SD	Median	Q1	Q3	IQR	Min	Max	Skewness	IQR outliers
	% total energy (provenance)	111	0	0.049	0.066	0.028	0.015	0.048	0.033	0.000	0.459	3.440	17

Descriptive statistics are presented for the dietary fat and fatty-acid variables examined in the study. Percentage-energy variables are reported as specified in the corresponding metric. *N*, number of observations; SD, standard deviation; Q1, first quartile; Q3, third quartile; IQR, interquartile range. Percentage-energy variables labelled as "provenance" represent the original Nutritics-derived values expressed relative to total energy. These are retained for data traceability and may differ slightly from the recalculated percentage-energy values excluding ethanol used for dietary-reference comparisons in the main analysis.

Table S4. Detailed distribution of dietary fat and fatty-acid intakes relative to UK dietary reference values.

Measure	Observed cohort statistic	UK reference value	Interpretation of reference	Cohort distribution relative to reference	Complementary classification
Total fat	mean 30.990 ± 5.180; median 30.568 [28.238–33.694]	35 % energy excluding ethanol	UK population-average DRV	at/below population benchmark: 95/111 (85.6%; 95% CI 77.9–90.9)	above population benchmark: 16/111 (14.4%; 95% CI 9.1–22.1)
Saturated fatty acids (SFA)	mean 11.129 ± 2.397; median 10.821 [9.830–12.640]	10 % energy excluding ethanol	UK population-average DRV	at/below population benchmark: 34/111 (30.6%; 95% CI 22.8–39.7)	above population benchmark: 77/111 (69.4%; 95% CI 60.3–77.2)
Total MUFA	mean 11.707 ± 2.581; median 11.309 [10.406–12.674]	12 % energy excluding ethanol	UK population-average DRV	—	—
Total n-6 PUFA	mean 2.119 ± 0.872; median 1.956 [1.652–2.326]	6 % energy excluding ethanol	UK population-average DRV	—	—
Alpha-linolenic acid (ALA, n-3)	mean 0.254 ± 0.111; median 0.246 [0.178–0.307]	0.2 % energy excluding ethanol	UK individual minimum	meets individual minimum: 75/111 (67.6%; 95% CI 58.4–75.6)	below individual minimum: 36/111 (32.4%; 95% CI 24.4–41.6)
Linoleic acid (LA, n-6)	mean 1.392 ± 0.635; median 1.321 [1.063–1.544]	1 % energy excluding ethanol	UK individual minimum	meets individual minimum: 89/111 (80.2%; 95% CI 71.8–86.5)	below individual minimum: 22/111 (19.8%; 95% CI 13.5–28.2)
Long-chain n-3 PUFA (EPA + DPA + DHA)	mean 0.241 ± 0.280; median 0.126 [0.081–0.254]	0.45 g/day	UK population-average DRV	at/above population benchmark: 19/111 (17.1%; 95% CI 11.2–25.2)	below population benchmark: 92/111 (82.9%; 95% CI 74.8–88.8)
Trans fatty acids	mean 0.486 ± 0.150; median 0.483 [0.382–0.562]	2 % energy excluding ethanol	UK population-average DRV	at/below population benchmark: 111/111 (100.0%; 95% CI 96.7–100.0)	above population benchmark: 0/111 (0.0%; 95% CI 0.0–3.3)
PUFA:SFA ratio	mean 0.451 ± 0.216; median 0.405 [0.334–0.506]	No formal threshold	Descriptive indicator	—	—
n-6:n-3 ratio	mean 4.120 ± 1.511; median 4.030 [3.164–4.983]	No formal threshold	Descriptive indicator	—	—
EPA + DHA	mean 0.206 ± 0.260; median 0.098 [0.061–0.209]	No formal threshold	Descriptive	—	—

Population-average dietary reference values are presented for population-level context and are not interpreted as individual requirements unless the source guidance explicitly defines an individual minimum. The observed MUFA variable represents total MUFA, whereas the 12% energy UK reference applies specifically to cis-MUFA; this comparison is therefore descriptive only. The n-6 population reference is likewise presented for descriptive population context.

Table S5. Adjusted differences in dietary fatty-acid intake between male and female participants (*N* = 111).

Outcome	Contrast	Adjusted difference, %	95% CI	*p*	BH *q*
Total fat (g/day)	Male vs Female	-10.53	-16.99 to -3.56	0.0040	0.0448
SFA (g/day)	Male vs Female	-13.67	-22.09 to -4.35	0.0054	0.0448
MUFA (g/day)	Male vs Female	-8.97	-16.91 to -0.27	0.0436	0.1484
PUFA (g/day)	Male vs Female	-16.29	-26.51 to -4.65	0.0079	0.0448
n-3 (g/day)	Male vs Female	-2.51	-21.77 to 21.50	0.8193	0.9269
n-6 (g/day)	Male vs Female	-6.34	-18.73 to 7.94	0.3622	0.8797
Trans (g/day)	Male vs Female	-0.69	-13.77 to 14.38	0.9231	0.9269
LA (g/day)	Male vs Female	3.22	-12.62 to 21.93	0.7070	0.9269
ALA (g/day)	Male vs Female	22.22	1.30 to 47.46	0.0364	0.1484
AA (g/day)	Male vs Female	14.00	-7.69 to 40.79	0.2211	0.6265
EPA (g/day)	Male vs Female	-13.62	-47.32 to 41.64	0.5584	0.9269
DPA (g/day)	Male vs Female	10.98	-16.38 to 47.27	0.4671	0.9269
DHA (g/day)	Male vs Female	-6.14	-47.19 to 66.82	0.8274	0.9269
PUFA:SFA ratio	Male vs Female	-3.04	-16.87 to 13.10	0.6921	0.9269
n-6:n-3 ratio	Male vs Female	-3.93	-18.44 to 13.17	0.6287	0.9269
EPA + DHA (g/day)	Male vs Female	-5.24	-42.65 to 56.56	0.8321	0.9269
EPA + DPA + DHA (g/day)	Male vs Female	-2.06	-37.42 to 53.30	0.9269	0.9269

Estimates were obtained from multivariable linear models of log-transformed outcomes adjusted for age, geographical background and log-transformed total energy intake. Heteroscedasticity-consistent HC3 standard errors were used. Effect estimates are expressed as the adjusted percentage difference in males relative to females. *q*-values were obtained using the Benjamini–Hochberg false-discovery-rate procedure. CI, confidence interval.

Table S6. Sensitivity analysis of adjusted sex differences after exclusion of participants in the sparsely represented geographical-background category (*N* = 109).

Outcome	Contrast	Adjusted difference, %	95% CI	*p*	BH *q*
Total fat (g/day)	Male vs Female	-9.75	-16.10 to -2.92	0.0063	0.0568
SFA (g/day)	Male vs Female	-13.64	-22.25 to -4.07	0.0067	0.0568
MUFA (g/day)	Male vs Female	-7.67	-15.12 to 0.44	0.0630	0.2141
PUFA (g/day)	Male vs Female	-15.20	-25.35 to -3.68	0.0117	0.0662
n-3 (g/day)	Male vs Female	-3.30	-22.68 to 20.94	0.7668	0.8374
n-6 (g/day)	Male vs Female	-4.81	-16.96 to 9.11	0.4752	0.8374
Trans (g/day)	Male vs Female	-2.05	-14.70 to 12.48	0.7674	0.8374
LA (g/day)	Male vs Female	3.61	-12.53 to 22.73	0.6789	0.8374
ALA (g/day)	Male vs Female	21.47	0.39 to 46.98	0.0456	0.1937
AA (g/day)	Male vs Female	9.65	-8.85 to 31.91	0.3249	0.8374
EPA (g/day)	Male vs Female	-18.12	-49.35 to 32.35	0.4109	0.8374
DPA (g/day)	Male vs Female	5.37	-17.69 to 34.89	0.6755	0.8374
DHA (g/day)	Male vs Female	-13.30	-49.54 to 48.96	0.6021	0.8374
PUFA:SFA (ratio)	Male vs Female	-1.82	-15.72 to 14.38	0.8124	0.8374
n-6:n-3 ratio	Male vs Female	-1.57	-15.49 to 14.64	0.8374	0.8374
EPA + DHA (g/day)	Male vs Female	-11.62	-44.84 to 41.60	0.6043	0.8374
EPA + DPA + DHA (g/day)	Male vs Female	-8.25	-39.51 to 39.17	0.6827	0.8374

Estimates were obtained using the statistical methods described in the main manuscript. HC3 heteroscedasticity-consistent standard errors were used for inference, and *q*-values were obtained using the Benjamini–Hochberg false-discovery-rate procedure, as applicable.

Table S7. Comparison of effect estimates from the primary and sensitivity analyses of sex differences in dietary fatty-acid intake.

Outcome	Primary estimate	Primary *q*	Sensitivity estimate	Sensitivity *q*	Same direction	Same FDR classification
Total fat (g/day)	-10.53	0.0448	-9.75	0.0568	True	False
SFA (g/day)	-13.67	0.0448	-13.64	0.0568	True	False
MUFA (g/day)	-8.97	0.1484	-7.67	0.2141	True	True
PUFA (g/day)	-16.29	0.0448	-15.20	0.0662	True	False
n-3 (g/day)	-2.51	0.9269	-3.30	0.8374	True	True
n-6 (g/day)	-6.34	0.8797	-4.81	0.8374	True	True
Trans (g/day)	-0.69	0.9269	-2.05	0.8374	True	True
LA (g/day)	3.22	0.9269	3.61	0.8374	True	True
ALA (g/day)	22.22	0.1484	21.47	0.1937	True	True
AA (g/day)	14.00	0.6265	9.65	0.8374	True	True
EPA (g/day)	-13.62	0.9269	-18.12	0.8374	True	True
DPA (g/day)	10.98	0.9269	5.37	0.8374	True	True
DHA (g/day)	-6.14	0.9269	-13.30	0.8374	True	True
PUFA:SFA (ratio)	-3.04	0.9269	-1.82	0.8374	True	True
n-6:n-3 ratio	-3.93	0.9269	-1.57	0.8374	True	True
EPA + DHA (g/day)	-5.24	0.9269	-11.62	0.8374	True	True
EPA + DPA + DHA (g/day)	-2.06	0.9269	-8.25	0.8374	True	True

Estimates were obtained using the statistical methods described in the main manuscript. HC3 heteroscedasticity-consistent standard errors were used for inference, and q-values were obtained using the Benjamini–Hochberg false-discovery-rate procedure, as applicable.

Table S8. Global tests of differences in dietary fatty-acid intake according to geographical background (*N* = 109).

Outcome	Robust Wald χ^2	df	Global *p*	BH *q*
Total fat (g/day)	7.755	2	0.0207	0.1058
SFA (g/day)	3.626	2	0.1632	0.3229
MUFA (g/day)	3.322	2	0.1900	0.3229
PUFA (g/day)	7.133	2	0.0282	0.1058
n-3 (g/day)	2.278	2	0.3201	0.4947
n-6 (g/day)	1.028	2	0.5982	0.6356
Trans (g/day)	6.187	2	0.0454	0.1101
LA (g/day)	3.449	2	0.1783	0.3229
ALA (g/day)	1.458	2	0.4823	0.5857
AA (g/day)	7.821	2	0.0200	0.1058
EPA (g/day)	0.605	2	0.7390	0.7390
DPA (g/day)	6.913	2	0.0315	0.1058
DHA (g/day)	1.790	2	0.4086	0.5498
PUFA:SFA (ratio)	8.419	2	0.0149	0.1058
n-6:n-3 ratio	6.575	2	0.0373	0.1058
EPA + DHA (g/day)	1.314	2	0.5185	0.5876
EPA + DPA + DHA (g/day)	1.733	2	0.4205	0.5498

Estimates were obtained using the statistical methods described in the main manuscript. HC3 heteroscedasticity-consistent standard errors were used for inference, and *q*-values were obtained using the Benjamini–Hochberg false-discovery-rate procedure, as applicable.

Table S9. Pairwise comparisons of dietary fatty-acid intake according to geographical background (*N* = 109).

Outcome	Contrast	Adjusted difference, %	95% CI	p	BH q
Total fat (g/day)	Africa vs Europe	-6.21	-14.31 to 2.66	0.1622	0.4354
	Asia vs Europe	3.61	-4.60 to 12.51	0.3963	0.6124
	Africa vs Asia	-9.48	-15.67 to -2.82	0.0064	0.1016
SFA (g/day)	Africa vs Europe	-4.76	-14.89 to 6.58	0.3922	0.6124
	Asia vs Europe	-9.78	-19.03 to 0.53	0.0621	0.3169
	Africa vs Asia	5.57	-4.70 to 16.94	0.2960	0.5536
MUFA (g/day)	Africa vs Europe	-7.68	-16.78 to 2.40	0.1293	0.4354
	Asia vs Europe	-0.95	-11.55 to 10.91	0.8673	0.9027
	Africa vs Asia	-6.80	-15.25 to 2.49	0.1448	0.4354
PUFA (g/day)	Africa vs Europe	-4.98	-18.50 to 10.78	0.5103	0.6705
	Asia vs Europe	13.46	-4.44 to 34.72	0.1476	0.4354
	Africa vs Asia	-16.26	-26.60 to -4.46	0.0088	0.1016
n-3 (g/day)	Africa vs Europe	13.20	-10.17 to 42.66	0.2901	0.5536
	Asia vs Europe	-3.74	-24.72 to 23.09	0.7592	0.8254
	Africa vs Asia	17.60	-6.22 to 47.47	0.1585	0.4354
n-6 (g/day)	Africa vs Europe	-8.62	-23.45 to 9.08	0.3148	0.5536
	Asia vs Europe	-6.75	-24.50 to 15.17	0.5127	0.6705
	Africa vs Asia	-2.00	-15.75 to 13.98	0.7911	0.8405
Trans (g/day)	Africa vs Europe	11.14	-5.64 to 30.90	0.2034	0.4666
	Asia vs Europe	-7.36	-21.61 to 9.48	0.3662	0.6025
	Africa vs Asia	19.97	3.68 to 38.82	0.0150	0.1275
LA (g/day)	Africa vs Europe	-16.69	-32.14 to 2.28	0.0804	0.3419
	Asia vs Europe	-16.05	-32.81 to 4.90	0.1225	0.4354
	Africa vs Asia	-0.76	-17.53 to 19.42	0.9347	0.9534
ALA (g/day)	Africa vs Europe	3.42	-16.87 to 28.66	0.7606	0.8254
	Asia vs Europe	-7.83	-27.77 to 17.60	0.5083	0.6705
	Africa vs Asia	12.21	-7.15 to 35.60	0.2304	0.4895
AA (g/day)	Africa vs Europe	-13.91	-31.98 to 8.97	0.2104	0.4666
	Asia vs Europe	-28.72	-44.10 to -9.12	0.0068	0.1016
	Africa vs Asia	20.78	-2.96 to 50.33	0.0900	0.3532
EPA (g/day)	Africa vs Europe	-8.59	-42.11 to 44.33	0.6973	0.8082
	Asia vs Europe	-17.80	-50.18 to 35.63	0.4393	0.6401
	Africa vs Asia	11.20	-32.81 to 84.04	0.6768	0.8027
DPA (g/day)	Africa vs Europe	-10.81	-28.65 to 11.49	0.3117	0.5536
	Asia vs Europe	-30.11	-46.68 to -8.39	0.0100	0.1016
	Africa vs Asia	27.62	-2.18 to 66.50	0.0718	0.3329
DHA (g/day)	Africa vs Europe	-11.35	-42.67 to 37.08	0.5848	0.7277
	Asia vs Europe	-31.18	-60.46 to 19.76	0.1839	0.4642

	Africa vs Asia	28.82	-25.07 to 121.47	0.3561	0.6025
PUFA:SFA (ratio)	Africa vs Europe	-0.24	-16.32 to 18.92	0.9784	0.9784
	Asia vs Europe	25.76	3.30 to 53.12	0.0229	0.1459
	Africa vs Asia	-20.68	-32.95 to -6.15	0.0074	0.1016
n-6:n-3 ratio	Africa vs Europe	-19.28	-32.47 to -3.51	0.0191	0.1392
	Asia vs Europe	-3.13	-19.36 to 16.35	0.7311	0.8254
	Africa vs Asia	-16.67	-30.55 to -0.01	0.0499	0.2826
EPA + DHA (g/day)	Africa vs Europe	-9.82	-40.70 to 37.12	0.6256	0.7597
	Asia vs Europe	-24.37	-53.37 to 22.64	0.2545	0.5191
	Africa vs Asia	19.24	-27.14 to 95.15	0.4803	0.6705
EPA + DPA + DHA (g/day)	Africa vs Europe	-9.65	-37.44 to 30.47	0.5850	0.7277
	Asia vs Europe	-24.55	-50.65 to 15.36	0.1911	0.4642
	Africa vs Asia	19.74	-22.85 to 85.86	0.4182	0.6273

Estimates were obtained using the statistical methods described in the main manuscript. HC3 heteroscedasticity-consistent standard errors were used for inference, and q-values were obtained using the Benjamini–Hochberg false-discovery-rate procedure, as applicable.

Table S10. Food-group contributions to dietary SFA, MUFA, PUFA and trans fatty-acid intake.

Fatty-acid class	Rank	Food group	Contribution, %	Bootstrap 95% CI	Participants, n (%)
SFA	1	Dairy foods & alternatives	19.7	17.2–22.0	109/111 (98.2%)
	2	Red & processed meat	18.7	16.4–21.1	110/111 (99.1%)
	3	Sweet discretionary, bakery & confectionery	16.6	13.9–19.7	109/111 (98.2%)
	4	Mixed/composite dishes & sauces	13.1	11.6–14.8	109/111 (98.2%)
	5	Added fats, oils, spreads & lipid-rich dressings	9.0	7.2–11.4	107/111 (96.4%)
	6	Fried potato/vegetable preparations	6.2	5.3–7.2	102/111 (91.9%)
	7	Cereals, breads & grains	3.3	2.8–3.9	111/111 (100.0%)
	8	Poultry	2.9	2.4–3.3	106/111 (95.5%)
	9	Fish & seafood	2.4	1.9–3.0	96/111 (86.5%)
	10	Savoury snacks	2.4	1.8–3.0	83/111 (74.8%)
	11	Nuts, seeds & nut/seed spreads	1.6	1.1–2.3	68/111 (61.3%)
	12	Fruit & non-fried vegetables	1.5	1.3–1.7	111/111 (100.0%)
	13	Eggs & egg dishes	1.5	1.1–1.8	95/111 (85.6%)
	14	Beverages & other low-fat foods	0.8	0.5–1.1	96/111 (86.5%)
	15	Pulses & plant-protein foods	0.3	0.2–0.4	103/111 (92.8%)
MUFA	1	Red & processed meat	18.3	16.1–20.5	110/111 (99.1%)
	2	Sweet discretionary, bakery & confectionery	14.7	12.3–17.3	109/111 (98.2%)
	3	Fried potato/vegetable preparations	11.9	9.4–15.1	102/111 (91.9%)

Fatty-acid class	Rank	Food group	Contribution, %	Bootstrap 95% CI	Participants, n (%)
	4	Mixed/composite dishes & sauces	9.7	8.6–11.2	108/111 (97.3%)
	5	Savoury snacks	8.7	6.9–10.7	83/111 (74.8%)
	6	Dairy foods & alternatives	7.7	6.7–8.8	109/111 (98.2%)
	7	Added fats, oils, spreads & lipid-rich dressings	6.1	5.1–7.1	107/111 (96.4%)
	8	Poultry	4.9	4.2–5.6	106/111 (95.5%)
	9	Fish & seafood	4.8	3.8–5.7	96/111 (86.5%)
	10	Nuts, seeds & nut/seed spreads	4.5	3.2–5.8	68/111 (61.3%)
	11	Cereals, breads & grains	4.1	3.6–4.7	111/111 (100.0%)
	12	Fruit & non-fried vegetables	2.1	1.7–2.7	111/111 (100.0%)
	13	Eggs & egg dishes	1.9	1.5–2.3	95/111 (85.6%)
	14	Beverages & other low-fat foods	0.3	0.2–0.5	94/111 (84.7%)
	15	Pulses & plant-protein foods	0.3	0.2–0.4	102/111 (91.9%)
PUFA	1	Sweet discretionary, bakery & confectionery	13.6	11.0–16.5	109/111 (98.2%)
	2	Fried potato/vegetable preparations	13.5	10.0–17.6	102/111 (91.9%)
	3	Red & processed meat	9.5	8.1–11.1	110/111 (99.1%)
	4	Fish & seafood	9.0	7.3–10.6	96/111 (86.5%)
	5	Nuts, seeds & nut/seed spreads	8.9	6.6–11.5	68/111 (61.3%)
	6	Cereals, breads & grains	8.8	7.7–9.9	111/111 (100.0%)
	7	Mixed/composite dishes & sauces	8.1	7.1–9.5	108/111 (97.3%)

Fatty-acid class	Rank	Food group	Contribution, %	Bootstrap 95% CI	Participants, n (%)
	8	Fruit & non-fried vegetables	5.4	4.8–6.0	111/111 (100.0%)
	9	Poultry	5.3	4.4–6.2	106/111 (95.5%)
	10	Added fats, oils, spreads & lipid-rich dressings	5.2	4.3–6.2	107/111 (96.4%)
	11	Savoury snacks	4.5	3.4–6.0	83/111 (74.8%)
	12	Dairy foods & alternatives	3.4	1.8–5.9	108/111 (97.3%)
	13	Pulses & plant-protein foods	2.4	1.7–3.3	103/111 (92.8%)
	14	Eggs & egg dishes	2.0	1.6–2.5	95/111 (85.6%)
	15	Beverages & other low-fat foods	0.3	0.2–0.4	94/111 (84.7%)
Trans	1	Red & processed meat	33.1	29.9–36.5	110/111 (99.1%)
	2	Dairy foods & alternatives	26.1	23.1–28.9	110/111 (99.1%)
	3	Mixed/composite dishes & sauces	11.8	10.3–13.4	108/111 (97.3%)
	4	Added fats, oils, spreads & lipid-rich dressings	9.0	6.7–11.8	96/111 (86.5%)
	5	Sweet discretionary, bakery & confectionery	7.4	6.1–8.8	109/111 (98.2%)
	6	Poultry	3.6	3.0–4.3	106/111 (95.5%)
	7	Fried potato/vegetable preparations	3.6	2.9–4.3	82/111 (73.9%)
	8	Cereals, breads & grains	2.4	1.8–3.1	105/111 (94.6%)
	9	Savoury snacks	1.8	1.2–2.5	76/111 (68.5%)
	10	Beverages & other low-fat foods	0.8	0.4–1.2	32/111 (28.8%)
	11	Fish & seafood	0.2	0.2–0.3	71/111 (64.0%)

Fatty-acid class	Rank	Food group	Contribution, %	Bootstrap 95% CI	Participants, n (%)
	12	Eggs & egg dishes	0.1	0.1–0.1	95/111 (85.6%)
	13	Pulses & plant-protein foods	0.0	0.0–0.0	12/111 (10.8%)
	14	Nuts, seeds & nut/seed spreads	0.0	0.0–0.0	55/111 (49.5%)
	15	Fruit & non-fried vegetables	0.0	0.0–0.0	0/111 (0.0%)

Contributions are expressed as percentages of reconstructed cohort-level intake and were calculated using a ratio-of-sums approach. Confidence intervals are participant-level percentile bootstrap 95% CIs based on 2,000 resamples. Participant n (%) indicates the number and proportion of participants with a positive reconstructed contribution to the corresponding fatty-acid class from foods assigned to that group. SFA, saturated fatty acids; MUFA, monounsaturated fatty acids; PUFA, polyunsaturated fatty acids; CI, confidence interval.

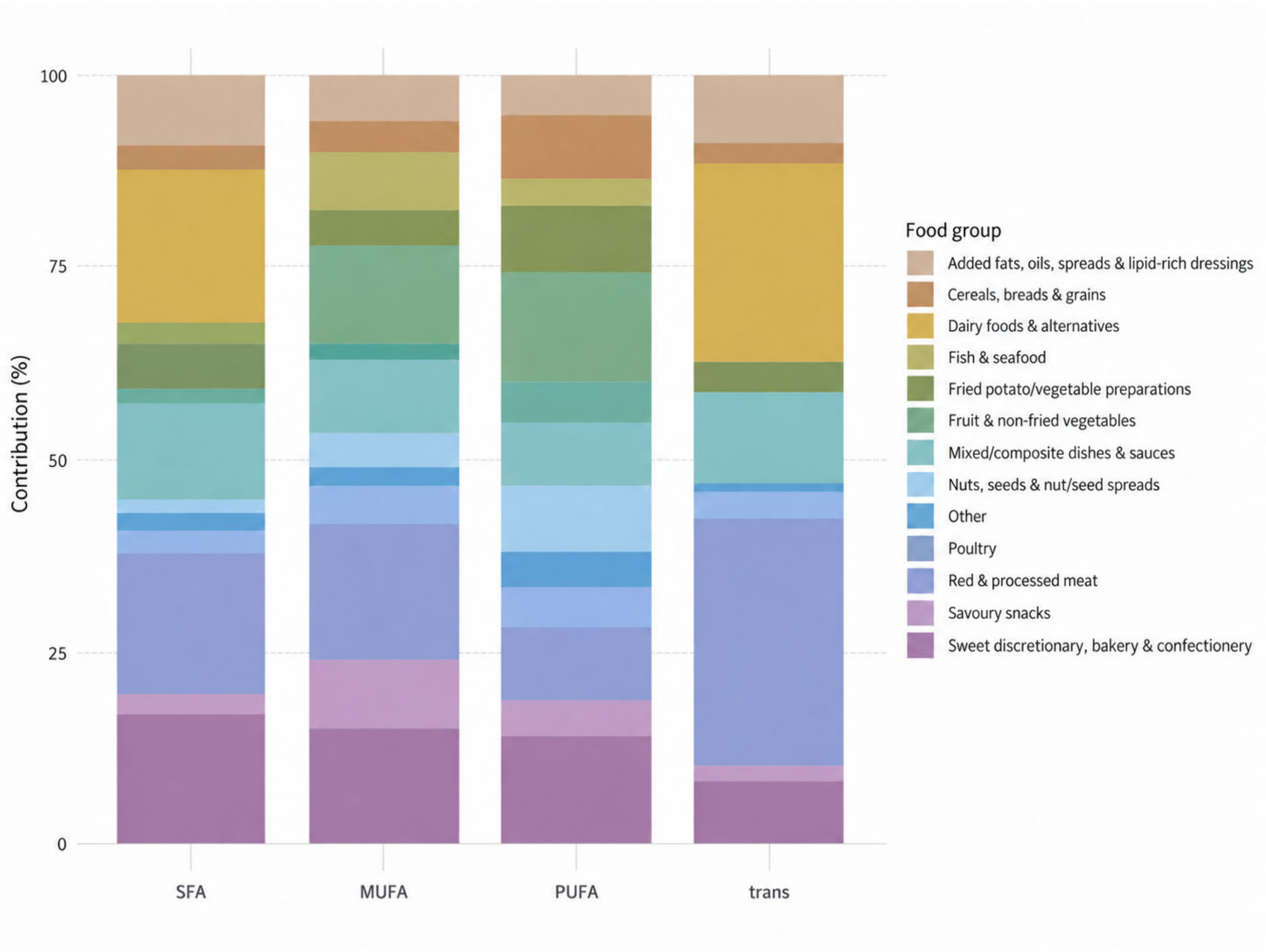

Figure S1. Complete food-group contribution profiles for dietary SFA, MUFA, PUFA and trans fatty acids. Contributions represent the percentage of reconstructed cohort-level intake attributable to each food group. SFA, saturated fatty acids; MUFA, monounsaturated fatty acids; PUFA, polyunsaturated fatty acids.
